# Dissecting the cellular architecture and genetic circuitry of the soybean seed

**DOI:** 10.1073/pnas.2416987121

**Published:** 2024-12-30

**Authors:** Julie M. Pelletier, Min Chen, Jer-Young Lin, Brandon Le, Ryan C. Kirkbride, Jungim Hur, Tina Wang, Shu-Heng Chang, Alexander Olson, Lachezar Nikolov, Robert B. Goldberg, John J. Harada

**Affiliations:** ^a^Department of Plant Biology, College of Biological Sciences, University of California, Davis, CA 95616; ^b^Department of Molecular, Cell, and Developmental Biology, University of California, Los Angeles, CA 90095

**Keywords:** embryo, endosperm, gene coexpression networks, seed coat

## Abstract

Seeds are complex structures composed of three regions, embryo, endosperm, and seed coat, with each further divided into subregions that consist of tissues, cell layers, and cell types. Although the seed is well characterized anatomically, much less is known about the genetic circuitry that dictates its spatial complexity. To address this issue, we profiled mRNAs from anatomically distinct seed subregions at several developmental stages. Analyses of these profiles showed that all subregions express similar diverse gene numbers and that the small gene numbers expressed subregion specifically provide information about the biological processes that occur in these seed compartments. In parallel, we profiled RNAs in individual nuclei and identified nuclei clusters representing distinct cell identities. Integrating single-nucleus RNA and subregion mRNA transcriptomes allowed most cell identities to be assigned to specific subregions and cell types and/or cell states. The number of cell identities exceeds the number of anatomically distinguishable cell types, emphasizing the spatial complexity of seeds. We defined gene coexpression networks that underlie distinct biological processes during seed development. We showed that network distribution among subregions and cell identities is highly variable. Some networks operate in single subregions and/or cell identities, and many coexpression networks operate in multiple subregions and/or cell identities. We also showed that single subregions and cell identities possess several networks. Together, our studies provide unique insights into the biological processes and genetic circuitry that underlie the spatial landscape of the seed.

## Significance

Seeds are a foundation of agriculture whose production is a key to global food security. Understanding the mechanisms that govern seed development may enable strategies to improve agricultural crops. To define the genetic circuitries that underlie the spatial complexity of seeds, we profiled gene activity in tissues and cell types of three seed regions- embryo, endosperm, and seed coat - and in individual nuclei from whole seeds. Data integration permitted the mapping of unique cell types and states spatially within the seed, the identification of biological processes that characterize cells and subregions, and the definition of gene networks that operate cell and subregion specifically and others that operate in several different cell types and subregions.

**Fig. 1.**
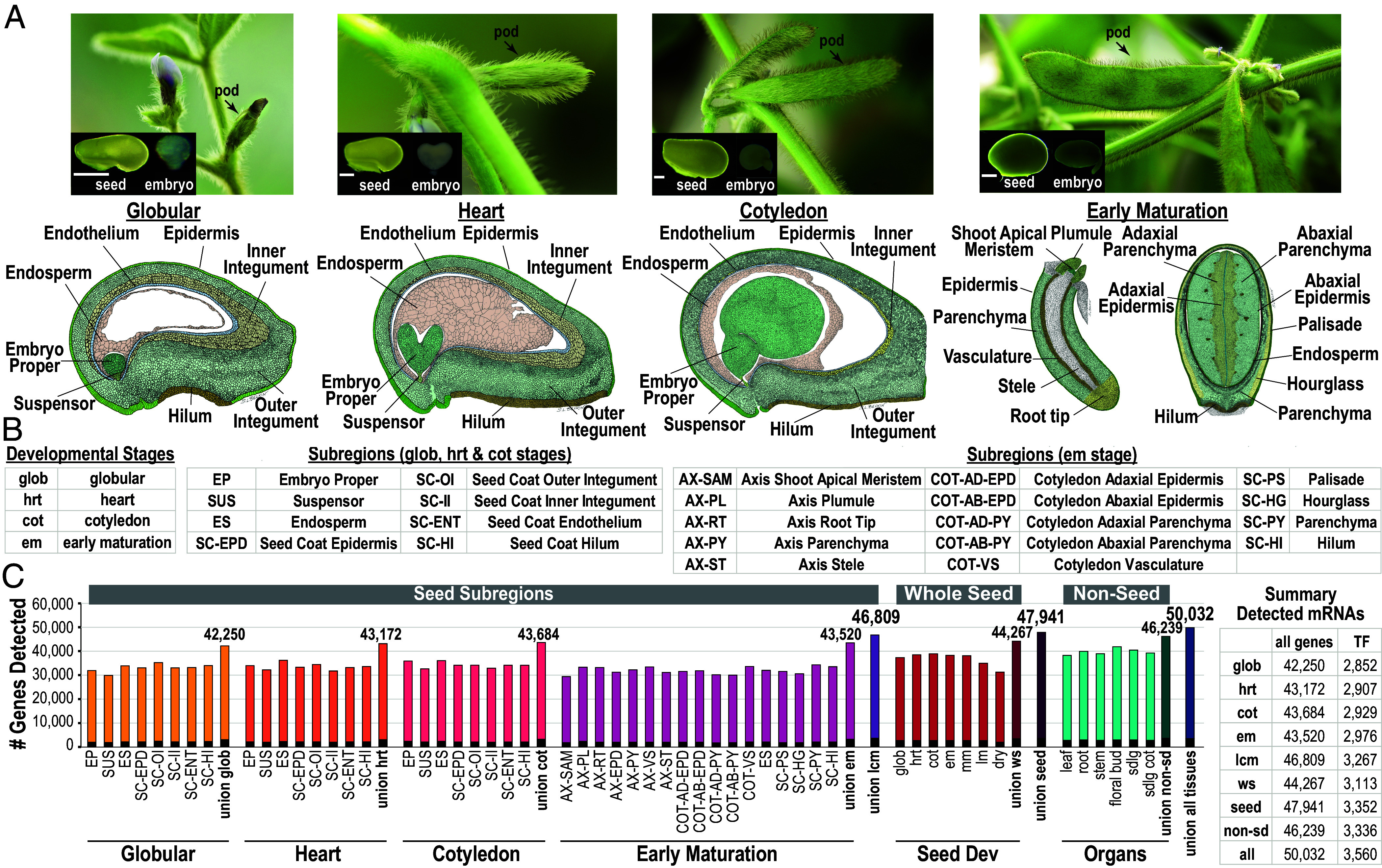
Genes active in regions, subregions, and tissues throughout soybean seed development. (*A, Top*) Pods, seeds, and embryos at the globular (glob), heart (hrt), cotyledon (cot), and early-maturation (em) stages. (Scale bars, 50 µm and 100 µm) at the glob, hrt, and cot stages and em stage, respectively. (*A, Bottom*) Drawings of longitudinal sections of glob, hrt, cot stage seeds, em stage embryo axis, and cross section of an em stage seed. (*B*) Seed subregions and developmental stages profiled using LCM, with abbreviations. (*C*) Number of diverse mRNAs, including those encoding transcription factors (TFs), in seed subregions, whole seeds, and vegetative organs throughout soybean development.

The angiosperm seed is an elegant developmental system, in part, because it is a complex structure composed of three distinct regions, embryo, endosperm, and seed coat. Each region is further compartmentalized into subregions, spatial domains that constitute the morphological and functional compartments of the seed. Subregions consist of one or more tissues and cell types, and, as shown for soybean seeds in Fig. 1, *A* and *B*, this complexity increases with developmental stage as continued differentiation gives rise to new tissues and cell types. The spatial complexity of the seed at the subregion, tissue, and cell-type level emphasizes that seed development is highly coordinated.

A comprehensive understanding of seed development requires knowledge of the biological processes that underlie the spatial complexity of the seed. We conducted studies to define the gene networks that govern seed development and to determine how these networks operate spatially within the seed. Gene networks describe the circuitry of coexpressed genes that mediate biological processes governing the structure, morphology, and physiology of cells, tissues, subregions, and regions of the seed. Defining the networks that operate in seeds and the subregions and cell types in which these networks operate is a key to obtaining a mechanistic understanding of seed development. Because networks operate at a cellular level, it is imperative that the cell types and cell states present throughout the seed be identified. Although much is known about seed structure (1, 2), recent single-cell sequencing studies have emphasized that the seed cellular composition is likely much more complex than thought previously, given that some cell identities cannot be detected anatomically (3–7).

Defining the gene networks that operate in regions, subregions, tissues, and cell types of the seed requires a comprehensive description of all expressed genes. mRNA transcriptomes have been profiled at the level of whole seeds (8–11), seed regions (12–15), and dissected subregions (16–18). However, most datasets lack the spatial resolution to assign gene networks to subregions and cells. To address this deficiency, we conducted experiments that describe the mRNA transcriptomes of dissected subregions and individual cells to define the gene networks that operate during soybean seed development. Integration of the single cell and subregion datasets permitted the assignment of cell identities to specific subregions and cell types and allowed us to define the networks that operate in each subregion and cell identity.

We show that the number of cell identities exceeds the number of anatomically distinguishable cell types, emphasizing the spatial complexity of seeds. Moreover, we show that gene networks exhibit a diversity of spatial distribution patterns in that some networks are specific to a single subregion and/or cell identity, whereas others operate in multiple subregions and cell identities. These studies provide unique insights into the genetic circuitry governing seed development.

## Results

### Experimental Strategy to Define the Gene Networks that Govern Seed Development and their Spatial Distribution in Subregions and Cell Types.

We used an integrated strategy to define the gene networks that underlie soybean seed development (*SI Appendix*, Fig. S1). Laser-capture microdissection was used to obtain RNA from dissected seed subregions and RNA sequencing (LCM–RNA Seq) to identify genes expressed in subregions throughout seed development. This method has been shown to isolate subregion RNA samples largely free of contamination from other subregions (16, 19).

In parallel, we isolated nuclei from cotyledon-stage seeds and used single-nucleus-RNA sequencing (snRNA Seq) to profile the snRNA transcriptomes of individual cells and to distinguish those with distinct identities. Integrating information obtained from these two experimental approaches allowed us to define the gene networks that operate in seeds and to identify the subregions and cell types in which these networks operate.

### Subregion mRNA Profiling Provides a Gene Expression Overview and Identifies mRNAs and Biological Processes Unique to Each Seed Subregion.

#### The extent of gene activity is similar in different seed subregions regardless of their biological complexity.

We profiled mRNA populations from the 41 seed subregions shown in Fig. 1, *A* and *B*, at the globular (glob), heart (hrt), cotyledon (cot), and early maturation (em) stages using LCM–RNA Seq (*SI Appendix*, Fig. S2 and Table S1). Fig. 1*C* shows that an average of 40,829 diverse mRNAs were detected in each subregion and that similar gene numbers were expressed in all seed subregions. By comparison, the soybean genome (Wm82.a2.v1) is predicted to contain 56,044 protein-coding genes. The biological complexity of a subregion did not correlate with the number of mRNAs detected. For example, the embryo and seed coat epidermises, each consisting of a single-cell layer, and the axis root tip, consisting of multiple cell types, all contained similar diverse mRNA numbers (Fig. 1*C*). In total, we detected 46,809 mRNAs in glob, hrt, cot, and em stage seeds using LCM-RNA Seq (Dataset S1), which is higher than the 44,267 diverse mRNAs detected in our RNA-Seq experiments using whole seeds, presumably because very low abundance mRNAs present in a subregion were detected by LCM but not whole seed analyses.

To estimate gene activity magnitude during seed development, we compared mRNA levels in each subregion (*SI Appendix*, Fig. S4, and Dataset S1). Although mRNA prevalence in a subregion varied over 5 to 6 logs, no substantial differences in the median mRNA prevalences of different subregions were detected. Characterization of transcription factor (TF) mRNA prevalences in different subregions and stages showed that there was no significant difference (*P* < 0.05) between the medians of TF mRNA versus all mRNAs in 15 of 41 subregions, and TF mRNA levels were only slightly below that of all mRNAs in the other subregions (*SI Appendix*, Fig. S4 and Table S2). Surprisingly, TF mRNA prevalence was broadly similar to that of all other mRNAs.

#### Similarities in mRNA transcriptome of subregions reflect their spatial organization in the seed.

We next examined the overall relationship between seed subregions by comparing subregion mRNA populations using hierarchical clustering and principal component analysis (*SI Appendix*, Fig. S5, *A* and B). Both analyses showed primarily that a subregion was more closely related to subregions in the same region than to those in other regions. For example, all seed coat subregions, regardless of stage, clustered together. Similarly, the EP and SUS that are both embryo compartments clustered together at the glob, hrt, and cot stages. One exception was that the glob, hrt, and cot EP mRNA populations were more closely related to ES subregions than embryo subregions at the em stage. This divergence likely reflects the major gene expression shift that occurs in embryo subregions as they transition from the morphogenesis to maturation phase of seed development (16). These findings suggest that subregions, even at different developmental stages, can be distinguished from other subregions on the basis of their mRNA transcriptomes. Therefore, gene expression patterns should provide specific information about the biological processes that occur in each subregion.

#### Small fractions of genes are expressed subregion specifically.

**Fig. 2.**
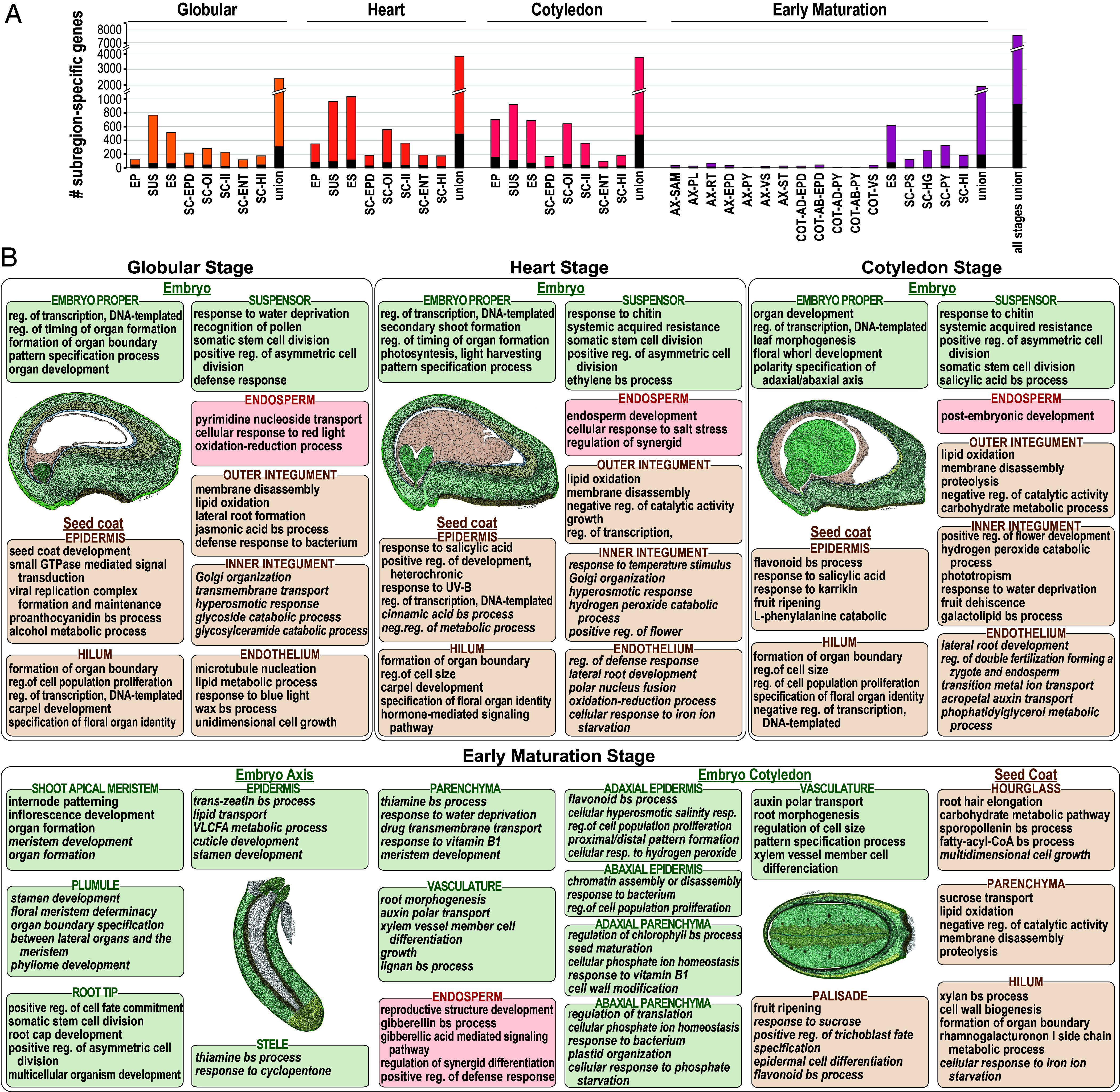
Specialization of gene expression and biological processes in seed subregions throughout development. (*A*) Number of subregion-specific mRNAs at each stage. (*B*) Top five biological process GO terms enriched for each subregion-specific mRNA set at each stage are listed (q < 0.05). Regional-specific GO terms (italicized) are given if fewer than five subregion-specific terms were identified. Drawings and abbreviations are as in Fig. 1.

Although there are overall similarities in subregion mRNA transcriptomes, we also identified genes expressed specifically in each subregion that likely play important roles in specifying subregion morphology and function. Fig. 2*A* shows that there were relatively few subregion-specific mRNAs, defined as those that accumulate at a fivefold or higher level (FDR < 0.001) in one subregion relative to all other subregions at the same stage. For example, an average of only 416 mRNAs accumulated subregion-specifically at the glob, hrt, and cot stages (Dataset S2). The median abundance of subregion-specific mRNAs was substantially higher than that of all mRNA, suggesting that the corresponding genes are relatively highly expressed (*SI Appendix*, Fig. S4 and Table S2). Even fewer subregion-specific mRNAs, averaging 105, were identified at the em stage because subregions were more highly dissected at this stage. Subregions within a region, especially the embryo, often share similar tissue types and, therefore, similar mRNA transcriptomes.

To accommodate similarities in subregion mRNA transcriptomes within the same region, we defined regional-specific mRNAs as those present in one subregion at a fivefold or higher level (FDR < 0.001) than other subregions in different seed regions at the same stage. The average number of regional-specific mRNAs, 978, was substantially higher than subregion-specific mRNAs, 416, and they provided a broader representation of genes that dictate subregion function (Dataset S2).

We identified subregion- and regional-specific TF mRNAs, given their likely regulatory importance in determining subregion function. *SI Appendix* Fig. S6 lists the most prevalent subregion/regional-specific TF mRNAs at different developmental stages. The median prevalence of subregion-specific TF mRNAs was generally higher than that of all TF mRNAs (*SI Appendix*, Fig. S4), suggesting that subregion-specific TF gene expression is relatively high.

#### Most mRNAs are shared among many seed subregions.

A corollary to our findings that each subregion expresses an average of 73% of genes in the genome and, on average, less than 1% of genes are expressed subregion-specifically is that most mRNAs in a subregion are shared among several different subregions. Forty-five percent of all mRNAs detected in seeds, 21,018, were detected in all subregions and stages profiled. Although these shared mRNAs are detected in many different subregions, they accumulate to different levels in different subregions. For example, the most abundant, non-subregion-specific mRNAs and TF mRNAs that are not subregion specific accumulated primarily in a single subregion or in related subregions (*SI Appendix*, Fig. S7). These mRNAs that accumulate primarily but not specifically in a subregion are likely to play important roles in mediating processes that contribute to the functions and morphology of that subregion.

#### Subregion-specific genes predict biological processes that occur uniquely in subregions.

Although seed subregions are well defined anatomically, little is known about the biological processes that characterize many subregions. To gain insight into the cellular processes that specify the unique morphology and function of a subregion, we determined GO term enrichment of subregion-specific and regional-specific mRNAs (Dataset S2). Fig. 2*B* shows an atlas of the biological processes predicted to be subregion specific. Many enriched biological processes correspond to those known to occur in subregions, such as meristem development in the shoot apex, root cap development in the root tip, cuticle development in the embryo epidermis, xylem vessel member cell differentiation in the embryo vasculature, seed maturation in the cotyledon adaxial parenchyma, and sucrose transport in the seed coat parenchyma. The analysis also provided unique information about understudied seed subregions. For example, hilum-specific GO terms shift from developmental processes to cell wall terms during the transition from cot to em stages, suggesting a functional transition from development to differentiation (Fig. 2*B*).

#### Seed development is conserved in soybean and Arabidopsis.

**Fig. 3.**
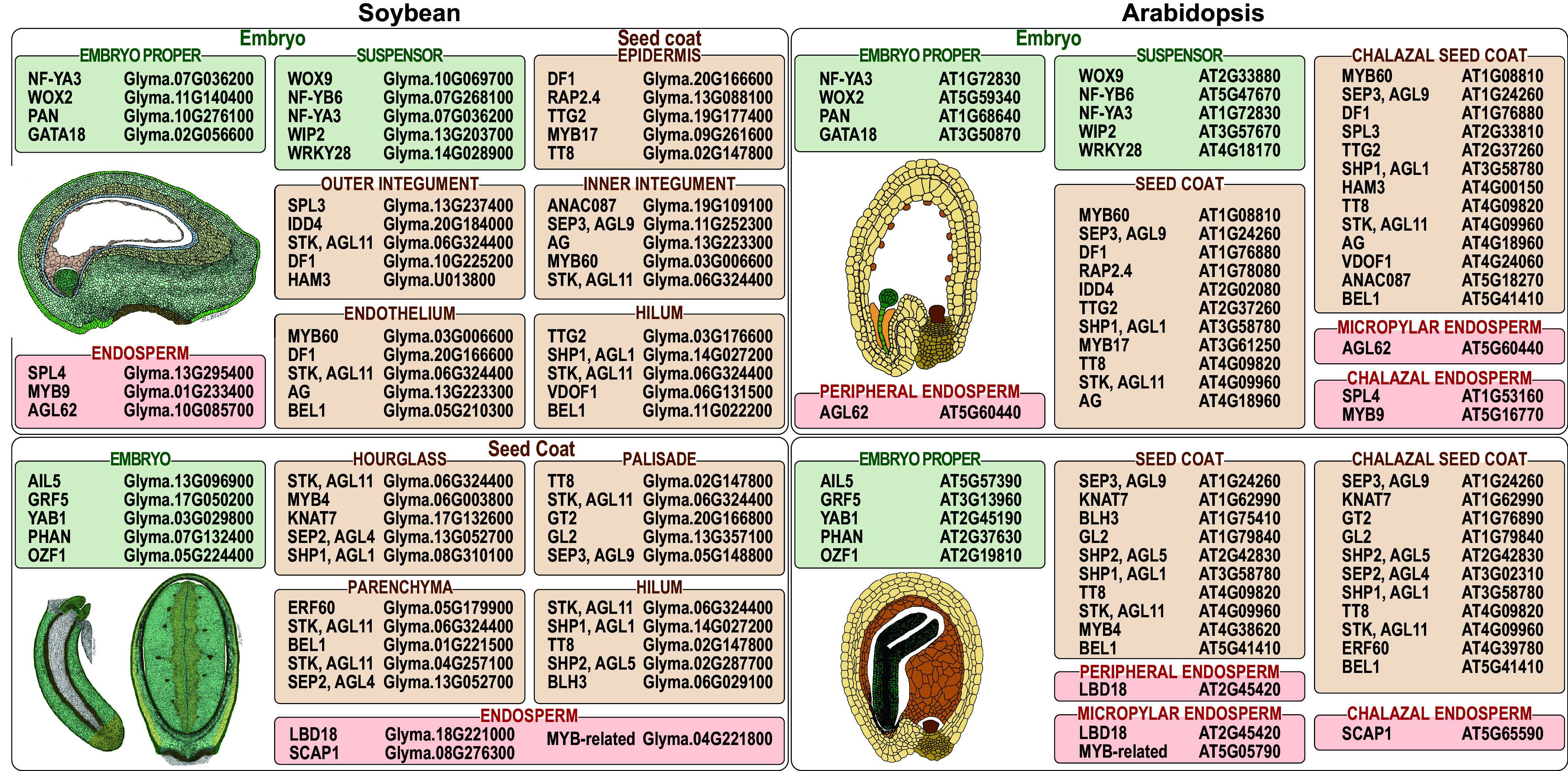
Conserved TFs specific to seed subregions in both soybean and Arabidopsis. Comparison of regional-specific TFs in soybean and Arabidopsis at the globular and early maturation/bent stages. The five most prevalent soybean regional-specific TFs that correspond to homologous regional-specific Arabidopsis TFs are listed.

Our previous study of gene activity in Arabidopsis subregions allowed us to directly compare soybean and Arabidopsis seed development (16). We first identified TF mRNAs that were regional-specific in both a soybean subregion and the corresponding Arabidopsis subregion. As shown in Fig. 3 and Dataset S2, we identified many TF mRNAs that are conserved between the two species. Many of these conserved TFs are known regulators of seed development, such as WOX2 and WOX9 in the embryo, AGL62 in the endosperm, and STK, TTG2, and TT8 in the seed coat (20, 21). We also asked whether biological processes in subregions are conserved in soybean and Arabidopsis and found that many GO terms that characterize seed coat subregions were shared. Of the 90 GO terms enriched for Arabidopsis distal and chalazal seed coat-specific mRNAs, 81% were enriched in at least one soybean seed coat subregion (*SI Appendix*, Fig. S8 and Dataset S2) (16). The finding that the same TFs and enriched GO terms characterize both soybean and Arabidopsis subregions suggests that gene regulatory networks and biological processes that occur in specific subregions are conserved during seed development in dicots.

### Single-Nucleus RNA Profiling Analysis Defines Distinct Cell Identities within Seed Subregions.

#### snRNA-Seq reveals the nuclear RNA transcriptome of single cells from cotyledon-stage seeds.

**Fig. 4.**
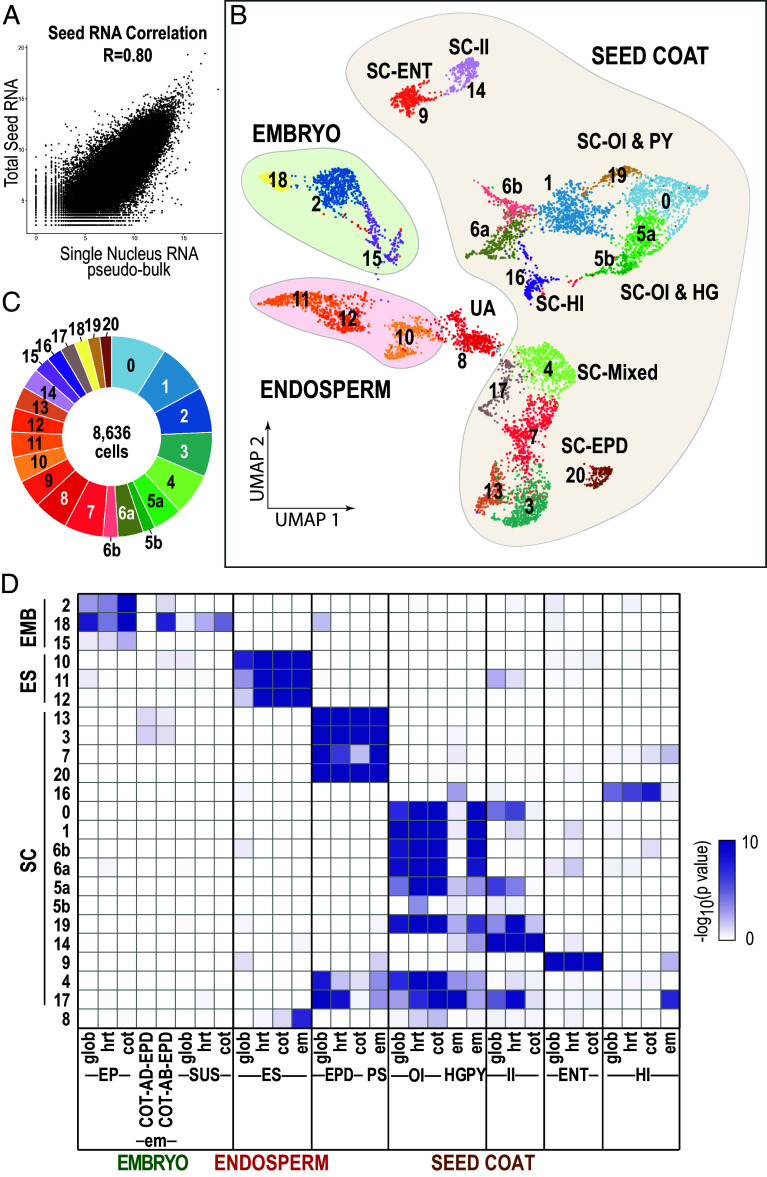
Single-nucleus RNA Seq of soybean cotyledon stage seeds defines multiple and distinct cell identities. (*A*) Scatter plot of RNA levels in whole seed total RNA and pseudobulk snRNA with Spearman's correlation coefficient. (*B*) Uniform Manifold Approximation and Projection (UMAP) dimensional reduction of cotyledon seed cells profiled and separated into 23 clusters. Dots represent cells, and colors identify distinct clusters annotated with their seed region or subregion of origin. (*C*) Proportion of cells in each cluster. (*D*) Enrichment of snRNA cluster markers for subregion-specific mRNAs. Clusters most highly enriched for subregion-specific mRNAs were assigned to that subregion.

Understanding how seed development is governed requires knowledge of the spatial distribution of networks within the seed. Because most subregions characterized in LCM-RNA Seq experiments consist of several distinct tissues and cell types, we used snRNA-Seq to define the transcriptomes of individual cells that constitute subregions (Dataset S3). Fig. 4*A* shows that the levels of total seed mRNAs and of the combined snRNA samples were highly correlated, indicating that RNA levels in the two samples exhibited strong similarities. Therefore, snRNA profiles appear to be a reasonable proxy for mRNA transcriptomes in cot-stage seeds.

We obtained quality snRNA profiles of 8,636 nuclei. As shown in Fig. 4, *B* and *C*, analyses of the data identified 23 snRNA clusters representing nuclei with highly similar snRNA transcriptomes (Dataset S4). To localize cells represented by clusters within the seed, we compared cluster marker RNAs—the most highly overrepresented snRNAs in each cluster—with subregion-specific mRNAs. Clusters were assigned to subregions with the highest enrichment for subregion-specific mRNAs. As shown in Fig. 4, *B* and *D*, the snRNA clusters mapped to all three seed regions and most subregions. Three clusters, defined by 1055 nuclei, mapped to the embryo. Clusters sn2 and sn15 corresponded to the EP, whereas sn18 mapped to the EP and SUS epidermises. Three endosperm clusters, sn10, sn11, and sn12, corresponded to 1053 nuclei. Sixteen seed coat clusters corresponded to 5,988 nuclei. Four mapped primarily to the SC-EPD (sn3, sn7, sn13, and sn20), seven to the outer integument (sn0, sn1, sn5a, sn5b, sn6a, sn6b, and sn19), two to the inner integument and endothelium, (sn14 and sn9), one to the hilum (sn16), and two mapped to multiple seed coat subregions (sn4 and sn17). One cluster corresponding to 496 nuclei, sn8, could not be assigned to a single seed region. Our ability to assign snRNA clusters to specific subregions based on LCM-RNA Seq data suggests a high level of correspondence between the two datasets. The high representation of seed coat clusters and low representation of embryo and endosperm clusters likely reflect relative cell numbers in each seed region and nuclei isolation efficiency from cot-stage seeds. For example, studies that specifically targeted Arabidopsis embryos and endosperm identified many more snRNA clusters from these regions than obtained here using whole seeds (3, 4).

#### snRNA transcriptomes permit identification of cell identities within subregions.

**Fig. 5.**
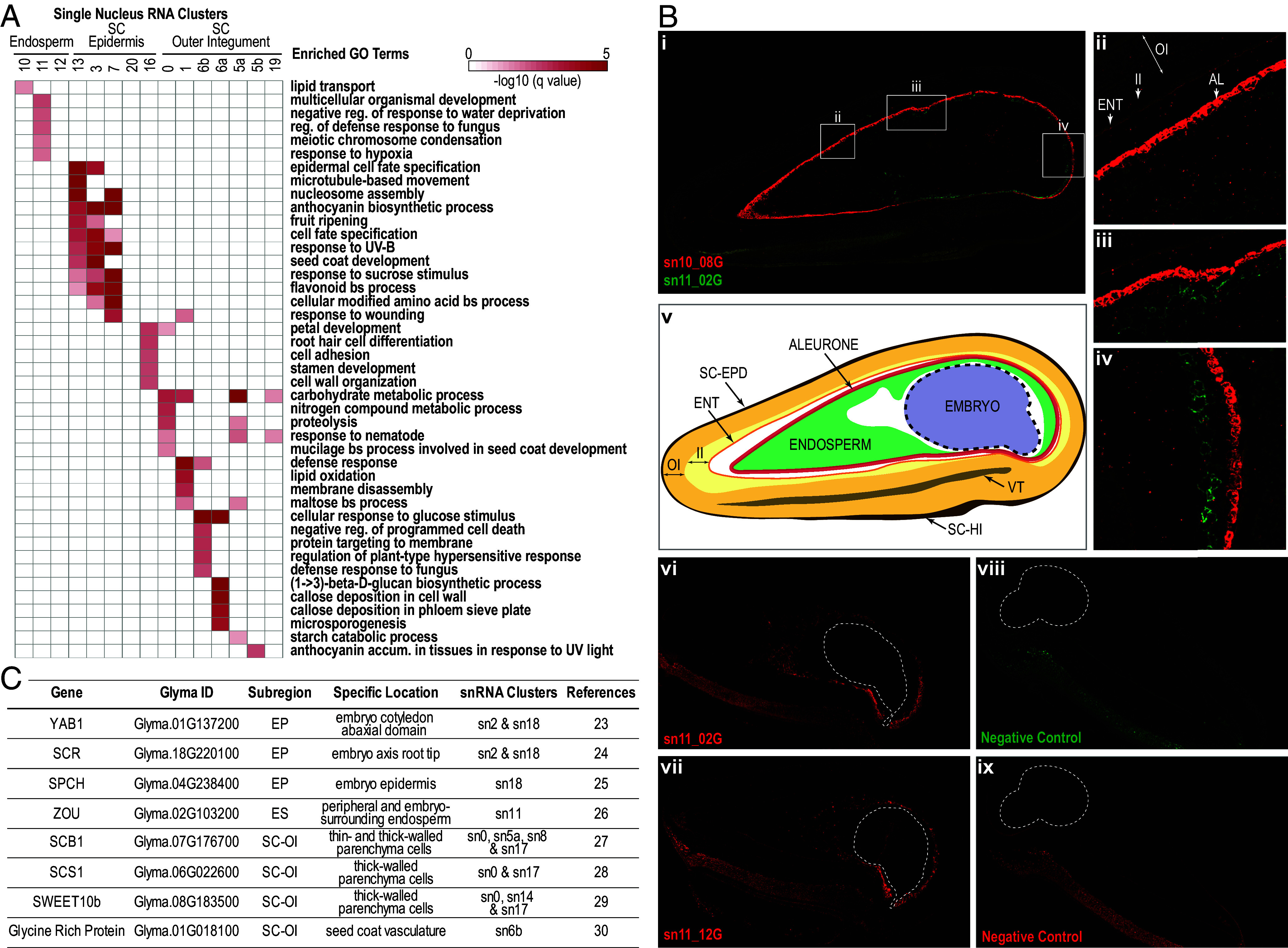
Different snRNA clusters from the same subregion correspond to distinct cell identities. (*A*) Heatmap showing that snRNA markers for different clusters representing endosperm, seed coat epidermis, and outer integument are significantly enriched for distinct GO terms. The top five enriched (FDR < 0.05) GO terms for each cluster are shown. (*B*) HCR localization of endosperm cluster marker mRNAs. *i–iv*) Confocal micrographs showing the localization of cluster 10-specific (sn10_08G) and cluster 11-specific (sn11_02G) mRNAs. *v*) Tracing of seed section shown in *i*) indicating seed subregions. *vi and vii*) Localization of two cluster 11-specific mRNAs, sn11_02G and sn11_12G, respectively. *viii and ix*) Negative controls. Signals from an amplifier with Alexa647 fluorophore are represented in red *(i and ix*, gain 750; panel *vi*, gain 700; panel *vii*, gain 610). Alexa546 signals are represented in green *(i–viii,* gain 500). Embryos in sections are outlined in white, dotted lines. (*C*) Reference mRNAs, their documented location in the seed, and their primary association with snRNA clusters.

Our finding that several snRNA clusters were associated with the same subregion suggested that these clusters correspond to cells with distinct identities. Consistent with this hypothesis, Fig. 5*A* shows that each cluster was enriched for distinct GO term sets, although there was some overlap of individual terms. Furthermore, the most prevalent snRNAs in each cluster differed (*SI Appendix*, Fig. S9), suggesting that genes in distinct clusters from the same subregion are differentially expressed.

**Fig. 6.**
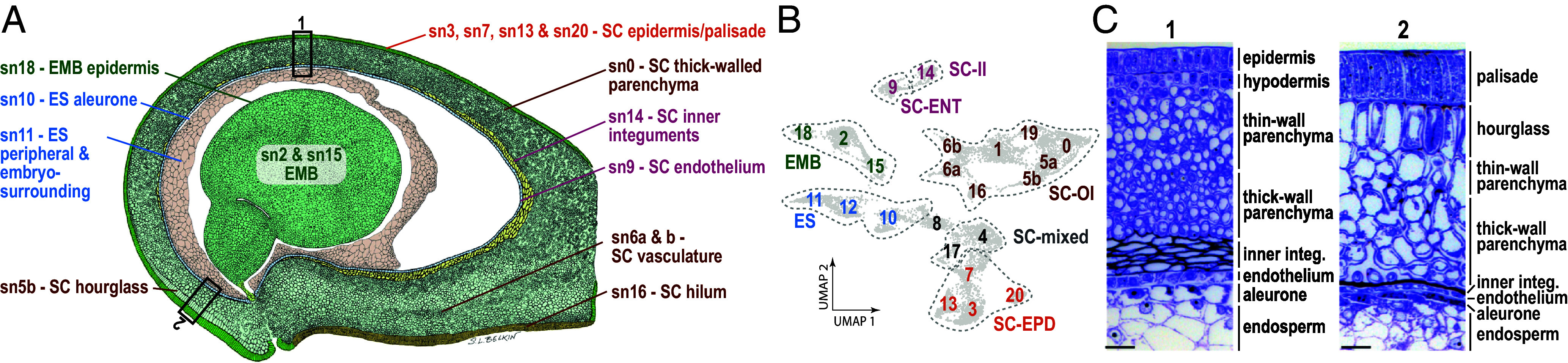
Cellular map of the soybean seed. (*A*) Illustration of a median longitudinal section through a cotyledon-stage seed annotated with snRNA clusters corresponding to seed cell types. (*B*) UMAP representation of cotyledon-stage seed snRNA clusters. (*C*) Toluidine blue O-stained plastic sections from the bottom *i*) and the top *ii*) of the cotyledon-stage seed coat. (Scale bar, 20 µm.)

We used several approaches to associate individual clusters with distinct cell types or states within a subregion. We first localized mRNAs specific for two endosperm snRNA clusters using Hybridization Chain Reaction (HCR) in situ hybridization experiments: a sn10 cluster marker mRNA, sn10_08G, and two sn11 cluster marker mRNAs, sn11_02G and sn11_12G, whose mRNA prevalences are shown in *SI Appendix* Fig S10. Fig. 5*B* shows that sn10_08G mRNA localized to the aleurone, a cell layer located at the endosperm periphery that consists of small, cytoplasm-rich, persistent cells with thick cell walls (1, 22) (Fig. 6). By contrast, sn11_02G and sn11_12G mRNAs were broadly localized to the embryo-surrounding and peripheral endosperm regions which consist of large, vacuolated cells with thin cell walls that are crushed as the embryo grows. Thus, we showed experimentally that sn10 and sn11 represent different cell identities that correspond to anatomically and developmentally distinct cell types.

We next examined the correlation between snRNA and subregion mRNA accumulation patterns (*SI Appendix*, Fig. S11*A*), comparison of GO term enrichment of snRNA cluster marker RNAs and subregion-specific mRNAs (Fig. 5*A* and Dataset S4), and the localization of reference mRNAs, whose position in the seed is documented in the literature (Fig. 5*C* and *SI Appendix*, Fig. S11*B*) (23–30), to associate embryo, endosperm, and seed coat snRNA clusters with specific cell types. For example, sn11 mRNAs were highly correlated with endosperm subregion mRNAs derived from LCM–RNA Seq experiments, consistent with the HCR experimental results. Moreover, snRNA for *ZOU*, a TF required for endosperm breakdown that is expressed in the embryo-surrounding and peripheral endosperm, accumulated primarily in sn11 nuclei (Fig. 5*C* and *SI Appendix*, Fig. S11*B*) (26).

These analyses allowed us to associate many snRNA clusters to specific cell types, as summarized in Fig. 6, *A* and *B*, particularly the seven distinct snRNA clusters in the seed coat outer integument which gives rise to hourglass and parenchyma cells. Cluster sn5b corresponds to hourglass cells based on marker mRNA accumulation patterns and subregion-specific mRNA enrichment (Fig. 4*D* and Dataset S4). Although a continuous hourglass layer is not discernable anatomically at the cot stage, Fig. 6, *A* and *C*, show that some hourglass cells are present in cot-stage seeds, consistent with the observation that a developmental gradient exists within the seed coat, with more developmentally advanced cells located at the hilum end of the seed (2, 31). This hypothesis is also consistent with the substantial overlap in the regional-specific mRNAs and GO terms between cot outer integument and em hourglass cells (Dataset S2), suggesting that some hourglass cells have differentiated prior to the em stage.

Four outer integument snRNA clusters, sn0, sn1, sn6a, and sn6b, were assigned to the seed coat parenchyma (Fig. 4*B*). The seed coat parenchyma is a complex subregion consisting of at least three tissues based on anatomical observations (2). The outer parenchyma layers consist of thin-walled parenchyma cells and vascular tissues, and the inner layers contain thick-walled cells (Fig. 6*C*). Two snRNA clusters, sn6a and sn6b, appear to be associated with vascular tissues. sn6a marker genes were enriched for GO terms related to vascular tissues, particularly phloem development, (Fig. 5*A* and Dataset S4), and a glycine-rich protein expressed specifically in vascular tissue maps to sn6b (Fig. 5*C* and *SI Appendix*, Fig. S11*B*) (30). Additionally, four *AtbZIP9* paralogs and two *SWEET13* paralogs that are expressed primarily in Arabidopsis phloem parenchyma cells were both sn6a and sn6b marker genes (Dataset S4) (32, 33). We infer that sn6a and sn6b cells represent distinct vascular, most likely phloem, cell types. Thick-walled parenchyma cells are marked by the expression of two genes, *SCS1* and *SWEET10b*, both of which mapped primarily to sn0 and sn17 (Fig. 5*C* and *SI Appendix*, Fig. S11*B*) (28, 29). Thus, sn0 cells are a strong candidate to represent the anatomically distinct thick-walled parenchyma cells (Fig. 6). The nature of sn17 cells is unclear, as they are also enriched for seed coat hourglass, epidermis, and hilum subregion-specific genes, opening the possibility that sn17 cells represent a progenitor of several different cell types.

It remains to be determined whether the other seed coat parenchyma cell identity, sn1, represents thin-walled parenchyma cell types or an alternative cell identity. Marker genes for the remaining outer integument clusters, sn5a and sn19, were expressed in both seed coat hourglass and parenchyma subregions. Thus, sn5a and sn19 cells may represent cell identities in developmental transition or morphologically indistinct cell types in the outer integument. We conclude that the snRNA-Seq studies identified at least three different outer integument cell types: hourglass, parenchyma vascular, and parenchyma thick-walled cells (Fig. 6).

Together, the snRNA Seq experiments emphasize the spatial complexity of seeds, with each subregion comprising multiple cell identities. Many of the cell identities were associated with anatomically distinguishable cell types, whereas others were not. The possibility exists that additional cell types and states are present in the seed.

### Gene Networks that Govern Biological Processes During Seed Development Operate Either Subregion/Cell Specifically or Across Multiple Subregions/Cell Identities.

#### Subregion mRNA profiles permit the identification of gene coexpression networks that operate in all three seed regions.

**Fig. 7.**
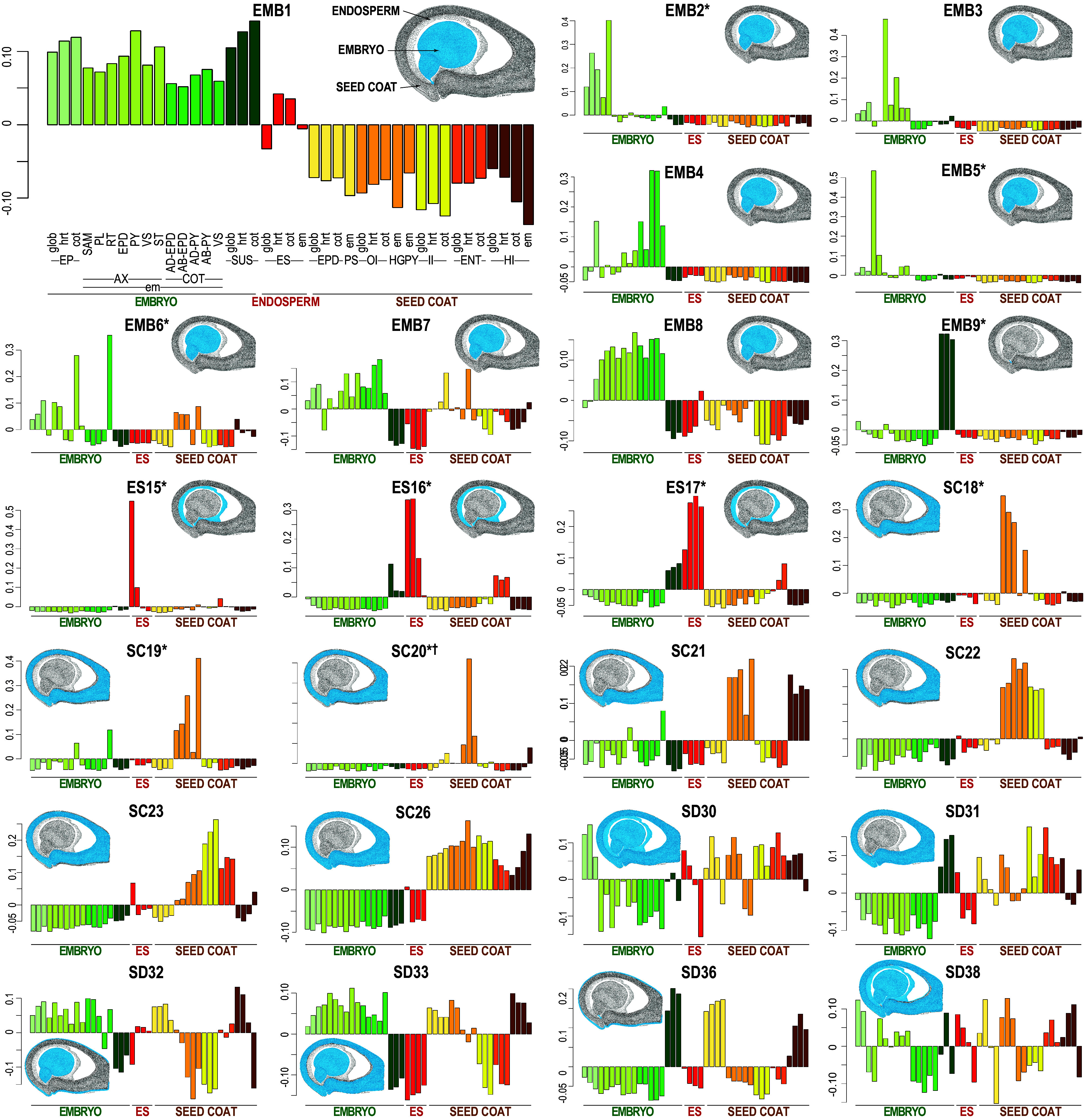
Gene coexpression analysis identifies networks that operate in different seed subregions. Module eigengene (representative mRNA levels) for 25 selected WGCNA modules that represent different coexpression networks. The *X* axis depicts average relative mRNA level, and the *Y* axis indicates subregions at different stages. Seed heat maps summarize module expression patterns. Subregion-specific modules are marked with an asterisk, and cell identity–specific modules are marked with a dagger.

**Fig. 8.**
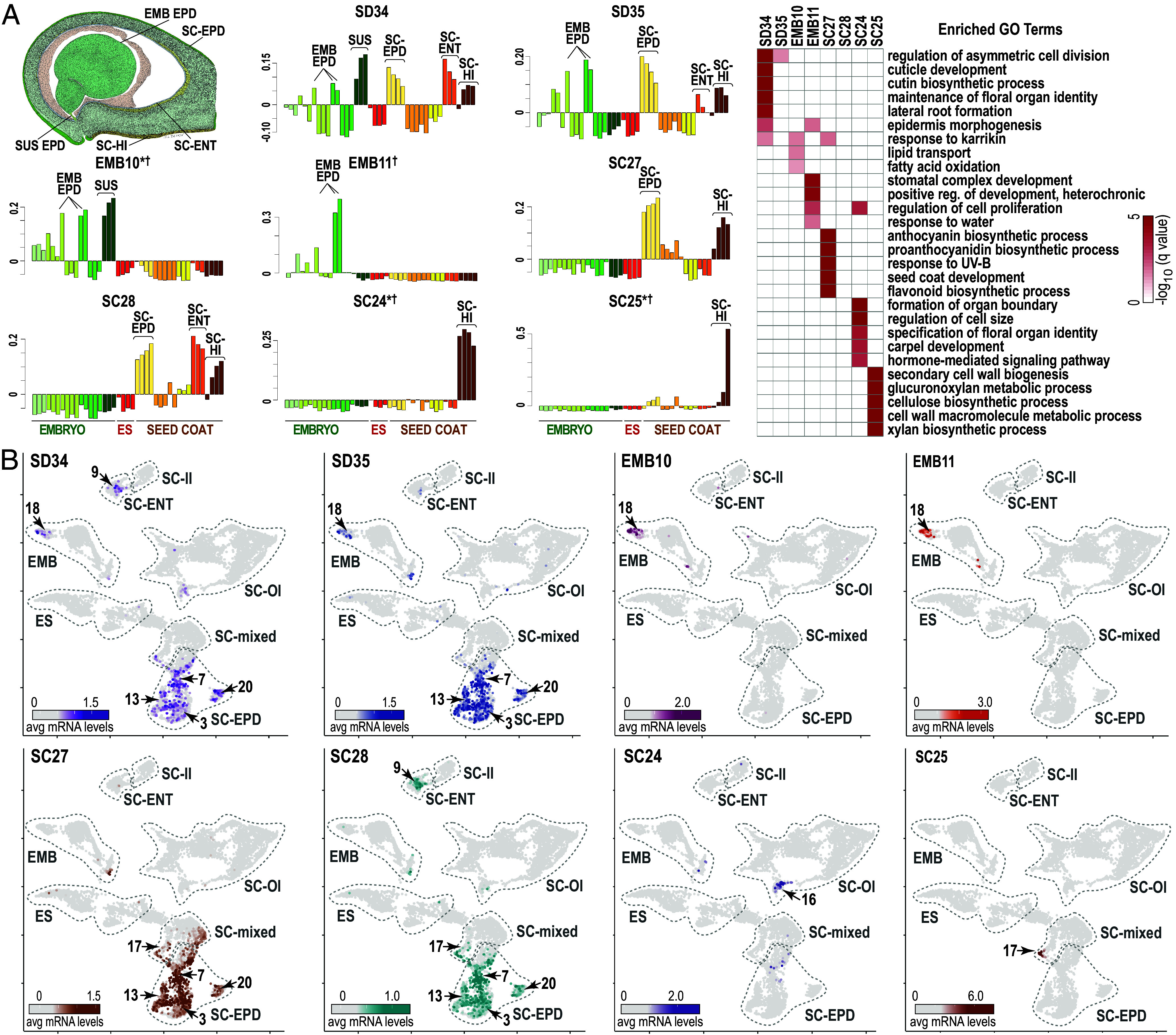
Common and specific coexpression networks operate in epidermal cell types. (*A*) Representative levels of epidermal module mRNAs that constitute common (SD34 and SD35) or specific (EMB10, EMB11, SC27, SC28, SC24, and SC25) coexpression networks. Seed epidermises are indicated on the cot-stage seed drawing. The heat map shows the significance of biological process GO term enrichment (FDR < 0.05) for the modules. The five most significant terms for each module are listed. Modules associated with a specific subregion or cell identity are marked with an asterisk or dagger, respectively. (*B*) Heatmap of the averaged snRNA levels of coexpression module mRNAs shown in (*A*) overlayed on the snRNA clusters. Clusters enriched for the indicated coexpression module mRNAs are numbered, and clusters corresponding to subregions are outlined (dotted line).

We used our seed subregion and cell transcriptome data to define the genetic circuitry that underlies the spatial complexity of the seed. We defined gene coexpression networks using the LCM–RNA Seq dataset because it provided more quantitatively accurate estimates of RNA levels than the snRNA Seq dataset, largely because of sequencing depth differences. This parameter is critical for defining coexpression networks. As shown in Figs. 7 and 8*A* and *SI Appendix*, Fig. S12, Weighted Gene Co-Expression Network Analysis identified 40 coexpression modules, each with an average of 231 mRNAs (Dataset S5) (34). All seed regions were represented by modules; mRNAs in 14, 3, and 12 modules accumulated primarily in the embryo (EMB), endosperm (ES), and seed coat (SC), respectively. The remaining 11 modules (SD) appeared to operate in multiple regions.

#### Many gene coexpression networks operate subregion and/or cell specifically.

We first focused on coexpression modules with high spatial specificity because subregion/cell-specific networks are important for tissue/cell-specific functions, presumably underlying biological processes that are unique to a single subregion and/or cell identity (35). We identified the 13 coexpression modules designated in Figs. 7 and 8*A* (Dataset S5) that appear to operate specifically in a single subregion. Consistent with this interpretation, an average of 77% of mRNAs in these modules were subregion-specific. Other mRNAs in these modules were enriched in the subregion but did not meet criteria to be designated as subregion specific. By comparison, only 20% of mRNAs in all other modules were subregion-specific. We also identified a few modules whose mRNAs appeared to map to a single-cell identity (designated in Figs. 7 and 8*A*). Examples of subregion/cell-specific networks include module EMB5 which is specific for the embryo shoot apex and is enriched for GO terms related to developmental processes, EMB6 which is specific for the embryo vasculature and other subregions containing developing vascular tissues and is enriched for vascular-related GO terms, and SC24 which is specific for a hilum cell identity (sn16) and enriched for developmental terms (*SI Appendix*, Fig. S13 and Dataset S5).

#### Most coexpression networks are shared across subregions and cell identities.

The majority of coexpression modules consisted of mRNAs that were not associated with a single subregion; all except modules EMB10, EMB11, SC20, SC24, and SC25 were not cell-identity specific. These modules appear to operate in multiple subregions and cell identities, indicating that the same biological processes mediated by the same gene sets occur in different subregions and cells. In support of this interpretation, the non-subregion-specific modules EMB4, EMB8, SD30, SD31, and SD38, respectively, were enriched for the following GO term sets: chloroplast and fatty acid synthesis; maturation, chloroplast, and gibberellin biosynthesis and signaling; photosynthesis and chloroplasts; stress and defense responses; and mitochondrial function (*SI Appendix*, Fig. S13 and Dataset S5). These biological processes are known to occur in many different subregions. A corollary of these observations is that most subregions and cell identities were associated with several coexpression networks. (Fig. 7)

#### Seed epidermises illustrate variability in network distribution among seed subregions and cell identities.

Epidermises are present in embryo axes and cotyledons, the outer seed coat layer, the inner seed coat, the endodermis, and the hilum, the attachment point between the seed and funiculus that consists of dual epidermal layers and vascular traces (2). As shown in Fig. 8*A*, we identified eight epidermis modules. mRNAs in two embryo modules, EMB11 and EMB10, accumulated primarily in the embryo proper and the embryo proper and suspensor, respectively. Four epidermal modules operated in the seed coat: SC27 in the seed coat epidermis and hilum, SC28 in the seed coat epidermis, hilum, and endothelium, and SC24 and SC25 exclusively in the hilum. These coexpression modules differentiate epidermises based on their spatial locations in the seed and, presumably, their functions.

To identify the cell identities in which the subregion-specific epidermal modules operate, we plotted the averaged snRNA level per cell onto the snRNA cluster map for the mRNAs present in each coexpression module. As shown in Fig. 8*B*, the two hilum modules, SC24 and SC25, mapped to two different clusters, sn16 and sn17, respectively, suggesting that each hilum network operates in distinct cell identities. By contrast, we found that many modules mapped to the same snRNA cluster. mRNAs for both embryo epidermis modules, EMB10 and EMB11, mapped to cluster sn18. Similarly, SC27 and SC28 mRNAs mapped to the same four seed coat epidermis snRNA clusters, sn3, sn7, sn13, and sn20. In addition, both of these seed coat epidermis modules mapped to sn17, whereas SC28 mapped to sn9. Thus, several distinct networks mapped to single-cell identities.

The biological processes predicted by GO term enrichment to be mediated by these networks provided support for these assignments. Both EMB10 and EMB11 modules were overrepresented for GO terms related to stomatal complex development and epidermis morphogenesis (Fig. 8*B*). Consistent with this interpretation, EMB11 contains three *GmSPCH* paralogs which we previously showed to be expressed specifically in cot and em-stage embryo epidermises and are required for meristemoid complex formation, a precursor of stomata (25). The seed coat epidermis SC27 module, sn3, sn13, sn7, and sn17 marker genes, and seed coat epidermis regional-specific genes at all developmental stages were enriched for GO terms related to the biosynthesis of anthocyanin and proanthocyanidin, compounds that accumulate in the seed coat epidermis and account for the black color of pigmented soybean (Fig. 8, *A* and *B*). Although the seed coat of the Wm82 cultivar used for these experiments is nonpigmented, other transcriptome experiments indicate that mRNAs encoding the vast majority of proanthocyanidin biosynthesis enzyme mRNAs accumulate similarly in seed coat epidermises regardless of whether the seeds are pigmented or not (36, 37). Thus, EMB10, EMB11, SC27, and SC28 coexpression networks, as well as most other modules, operate in several distinct cell identities (Fig. 8*B* and Dataset S5).

In addition to these region-specific networks, we identified two modules that were associated with most all epidermises. SD35 mRNAs mapped to the embryo axis and cotyledon epidermises, and seed coat epidermis, endothelium, and hilum, whereas SD34 mRNAs mapped to all of these subregions and the SUS (Fig. 8*B*). Similarly, we showed that SD34 and SD35 mRNAs mapped to all epidermal snRNA clusters, except the hilum cluster, sn16. Given that networks conserved across tissues are likely to have functions common to those tissues (35), SD34 and SD35 may underlie general epidermal functions regardless of their seed location. Plant epidermises generally share common features, such as a thicker wall on the external face of the cell that provides mechanical strength to an organ and a lipid-rich cuticle in the external cell matrix that prevents the penetration of water, aqueous molecules, and pathogens through the epidermis (38, 39). Consistent with general epidermis function, SD34 was enriched for GO terms related to lipid and cuticle biogenesis, although SD35 was not enriched for GO terms. The seed epidermises illustrate that coexpression modules may be associated with a single-cell identity/subregion or with multiple cell identities/subregions. Thus, some modules are associated with biological processes unique to a subregion/cell identity, whereas other modules are associated with biological processes that occur in several different cell identities/subregions.

## Discussion

To obtain a comprehensive understanding of seed biology, we profiled mRNAs in regions, subregions, and cells throughout development. Gene coexpression networks defined with the mRNA profiles provided mechanistic information about the processes that underlie seed development. Our analyses also described the snRNA profiles of specific cell identities within seeds and indicated which gene networks operate in specific subregions and cells. These analyses provide unique insights into the biological processes that govern seed development and the spatial distribution of gene networks within the developing seed.

### mRNA and snRNA Profiles Enable Dissection of the Cellular Landscape of the Seed.

#### Gene expression programs reflect the ontogenetic origin of subregions.

Our finding that seed subregions are more closely related to those from the same rather than other regions demonstrated that there is greater similarity among subregions of filial origin, embryo and endosperm than between the filial and maternal seed coat subregions (*SI Appendix*, Fig. S5, *A* and B). These relationships are consistent with the hypothesis that the endosperm derived from an ancient secondary embryo and, therefore, that the embryo and endosperm of angiosperms share a common evolutionary origin (40). Similar conclusions were obtained from studies of Arabidopsis and maize seed mRNA transcriptomes, emphasizing the conservation of seed development (16, 18).

#### Integration of the LCM and snRNA datasets defines the cellular constituents of seed subregions.

We profiled the snRNA populations of distinct cell identities to establish their relationship to seed subregions and to determine where gene networks operate in the seed at high spatial resolution. Assignment of cell clusters from cot-stage seeds established that multiple cell identities are associated with each subregion (Fig. 4). Cell identities defined in single-cell sequencing analyses generally correspond to cells of different types or states (41, 42). Cell types are often distinguished by morphological, physiological, functional, and/or molecular characteristics, whereas cell states can represent variations of a cell type resulting from differences in cell cycle phase, responses to the environment, or developmental transitions from one type to another (43, 44).

Our assignment of cot-stage seed cell identities to specific types or states within subregions is summarized in Fig. 6. In many cases, the different cell identities correspond to anatomically distinct cell types. For example, one endosperm cell identity, sn10, corresponds to aleurone, whereas sn11 corresponds to the embryo surrounding and peripheral endosperm. The seed coat outer integument is a subregion in which cluster assignments as cell types and states are ambiguous. sn5b, sn6a and sn6b, and sn0 cell identities correspond to seed coat hourglass, vascular parenchyma, and thick-walled parenchyma cells, respectively. By contrast, sn5a, sn17, and sn19 clusters map to several different subregions, making it unclear whether these cell identities represent unique cell types or states. Similarly, sn3, sn13, sn7, and sn10 all map to the seed coat epidermis, but it is unclear whether these cell identities correspond to different cell types or states. Nevertheless, the analysis provides a unique description of the transcriptomes of specific seed cell types and cell states. This information was critical in allowing us to determine which networks operate in specific cell types and states.

### Comprehensive Analyses of Gene Expression in Developing Seeds Indicate that Gene Networks Exhibit Diverse Spatial Distribution Patterns.

#### Overview of gene expression in developing seeds.

The RNA profiles of seed regions, subregions, tissues, and individual nuclei indicated that ~85% of all protein coding genes in the genome are expressed at some point during seed development. All subregions express similar numbers of genes, and only ~1% of all diverse mRNAs are expressed subregion-specifically (Figs. 1 and 2). These findings indicate that the extent and specificity of gene expression in a subregion does not reflect the biological complexity of tissues and cell types. They further suggest that differentially expressed genes that are shared among subregions in combination with subregion-specific genes, likely play key roles in distinguishing the unique morphology and function of each subregion.

Our analysis has provided information about gene expression levels during development. Despite wide variations in the levels of diverse mRNAs in each subregion, the median mRNA prevalence of all subregions is similar (*SI Appendix*, Fig. S3). Counter to general expectations that TF genes are expressed at low levels, we found TF mRNA prevalence to be only slightly lower than that of all mRNAs (*SI Appendix*, Fig. S4 and Table S2). By contrast, subregion-specific mRNAs, including those encoding TFs, are generally present at a higher level than that of most other mRNAs. Similar conclusions about the extent and levels of gene expression in soybean seeds derive from other mRNA transcriptome studies and from classical mRNA-single copy DNA reassociation experiments (10, 45).

Finally, comparison of developing soybean and Arabidopsis seeds mRNA transcriptomes suggests that the many cellular processes that occur during seed development are conserved. Both soybean and Arabidopsis share regional-specific TFs, and an average of 19% of conserved TFs are components of soybean seed coexpression modules, suggesting their regulatory roles in gene networks (Fig. 3 and Dataset S2). Many of the conserved TFs are known key regulators of seed development and/or hub genes in coexpression networks, indicating their importance in the seed genetic circuitry (Fig. 3 and Dataset S5). Similarly, there was significant overlap in enriched GO terms in the seed coat subregions of soybean and Arabidopsis (*SI Appendix*, Fig. S8), although we did not detect overlap in the embryo and endosperm. Thus, there is considerable conservation in the seed gene networks that operate in soybean and Arabidopsis, even though they diverged approximately 92 mya (46). The fact that both plants are oilseeds and, therefore, share overlapping morphology and physiological processes, likely accounts for the genetic conservation.

#### Gene networks governing seed development exhibit diverse spatial distribution patterns.

We defined gene coexpression networks to obtain a comprehensive understanding of the genetic circuitry involved in the control of seed development. GO term enrichment of coexpression module mRNAs also provided insight into the biological processes with which the networks are involved. Localizing the expression of module genes to subregions and cell identities helped to define how these networks operate spatially in the seed and established the following points. First, many coexpression networks are specific to a single subregion and/or cell identity (Figs. 7 and 8 and Dataset S5). These networks presumably underlie processes that are unique to a single subregion and/or cell identity (Figs. 7 and 8 and *SI Appendix*, Fig. S13 and Dataset S5). Second, most coexpression networks are not linked to a single subregion or cell identity. This indicates that many gene networks operate in multiple subregions and cell identities, suggesting that the same genes and the same biological processes, such as photosynthesis, maturation, and stress responses, occur in many different cells, subregions, and regions of the seed. Third, most subregions and cell identities are associated with several modules. Numerous networks are required to mediate the constellation of biological processes that occur in a subregion or cell identity. It is likely that other networks operate in subregions and cell identities despite the very high resolution of our datasets.

## Conclusion

We provide a comprehensive map of gene activity in the soybean seed throughout its development. Analyses of mRNA profiles of embryo, endosperm, and seed coat subregions and of snRNA profiles of cells from cot-stage seeds enabled the identification of many cell types and states that populate seed subregions. The analyses enabled the prediction of the gene networks that operate in seed regions, subregions, and cell types and states which in turn permitted the biological processes that occur subregion and/or cell specifically or across several different subregions to be predicted. Our studies provide foundational information that will be useful in defining the development and evolution of specific cell types and in understanding the genetic basis for their unique physiological and morphological functions. Given that seeds account for a large fraction of the calories consumed by humans, this fundamental information may also be useful for the design of strategies needed to improve crops for agriculture.

## Materials and Methods

### Plant Materials, Growth Conditions, Tissue Collection, and LCM.

Soybean cv Williams 82 plants were grown, seeds were staged, samples were prepared for LCM, and sequencing libraries were prepared as described (16, 47–50) with modifications listed in *SI Appendix, Supporting Information Materials and Methods*.

### Nuclei Isolation and Single-Nucleus Sequencing.

Nuclei were isolated from 50 cotyledon-stage seeds using the following protocol (51) with the modifications described in SI Materials and Methods. Single-nucleus RNA and ATAC Seq libraries were prepared with the Chromium Single-Cell Multiome ATAC + Gene Expression Assay (10X Genomics, V1), according to the manufacturer's instructions. Libraries were sequenced on a NovaSeq 6000 Instrument, targeting 20,000 and 25,000 read pairs per nuclei for RNA- and ATAC-Seq, respectively. Only snRNA-Seq results are presented.

### HCR In Situ Hybridization.

Soybean cotyledon-stage seeds were fixed, processed, and sectioned as described (16, 48) and mounted on Probe-On Plus slides (Fisher Scientific, 22-230-900), incubated at 42°C overnight, and stored at room temperature.

Hybridization probes with split-initiators targeting specific transcripts and fluorescent amplifying hairpins (Alexa Fluor 546, or 647) were designed and purchased from Molecular Instruments. HCR RNA–FISH was performed according to (52), and the Molecular Instruments protocol (https://files.molecularinstruments.com/MI-Protocol-RNAFISH-FFPETissue-Rev4.pdf) with modifications described in SI Materials and Methods. Sections were imaged using a Zeiss LSM710 confocal microscope.

### LCM Data Analysis.

Raw sequencing data from LCM and whole mount samples were processed as described (16, 48). Arabidopsis LCM data were published previously (16, 49). Detected mRNAs in the LCM subregions had at least one raw count in each biological replicate. In the whole seed dataset, detected mRNAs have CPM greater than 0.0978 (average CPM equivalent to one raw count in the LCM subregions). GO enrichment analyses were performed as described (49). We used the R package WGCNA (34, 53) to identify modules of coexpressed genes in the LCM dataset. Additional details are provided in SI Materials and Methods.

### Single–Nucleus RNA Seq Analyses.

Base calls from MultiOme raw data were processed into fastq files using Illumina bcl2fastq v2.20.0.422, and raw files were analyzed by Cell Ranger ARC v1.0. Soybean genome assembly v2.0 and Gmax_275_Wm82.a2.v1.gene_exons.gff3 were downloaded from Phytozome (https://phytozome-next.jgi.doe.gov) and used to create a custom reference genome using Cell Ranger ARC mkref function. Single-cell feature counts were generated using the count function. Detailed run metrics are presented in Dataset S3.

Ambient RNA contamination was removed using the R package SoupX v1.6.1 using default parameters (54). The cleaned snRNA count matrix was imported to Seurat v4.1.1 (55) for downstream analyses. We used SCTransform normalization (56), performed dimension reduction using Principal Component Analysis and UMAP embedding, and clustered cells using FindNeighbors (dims = 30) and FindClusters (dims = 30, resolution = 1.0). FindSubCluster with appropriate resolution was used to divide some clusters in two. Data were renormalized after subclustering using the “RNA” pipeline and cluster markers were identified using FindAllMarkers (only.pos = T, min.pct = 0.25). Cluster markers were further filtered for log2FC >= 1 and adjusted *P* value < 0.05.

snRNA clusters were assigned to seed subregions by comparing cluster markers to subregion-specific mRNAs for the cotyledon and early maturation stages, using the GeneOverlap R package (57). Bulk RNA-Seq and pseudobulk snRNA-Seq values were compared as in ref. 58 using Spearman rank correlation.

## Supplementary Material

Appendix 01 (PDF)

Dataset S01 (TXT)

Dataset S02 (XLSX)

Dataset S03 (XLSX)

Dataset S04 (XLSX)

Dataset S05 (XLSX)

## Data Availability

The LCM seed subregion and cotyledon stage single-nucleus RNA datasets were deposited in GEO, Accession Nos: GSE116036 (59) and GSE243174 (60), respectively. All other data are included in the manuscript and/or supporting information.
