# Supplementary material for: Dissecting the cellular architecture and genetic circuitry of the soybean seed
Source: Proc Natl Acad Sci U S A. 2024 Dec 30;122(1):e2416987121. doi: 10.1073/pnas.2416987121 (PMC11725896; doi:10.1073/pnas.2416987121)
Supplement: Supplementary file 1 — Appendix 01 (PDF) [file pnas.2416987121.sapp.pdf]

PNAS

Supporting Information for
Dissecting the Cellular Architecture and Genetic Circuitry of the Soybean Seed.

Julie M. Pelletier, Min Chen, Jer-Young Lin, Brandon Le, Ryan C. Kirkbride, Jungim Hur, Tina Wang, Shu-Heng Chang, Alexander Olson, Lachezar Nikolov, Robert B. Goldberg, John J. Harada.

Robert B. Goldberg
Email: bobg@ucla.edu

John J. Harada
Email: jjharada@ucdavis.edu

This PDF file includes:

Supporting text
Figures S1 to S13
Table S1
Table S2
SI References

Other supporting materials for this manuscript include the following:

Datasets S1 to S5

Supporting Information Materials and Methods

LCM Libraries. LCM RNA-seq libraries were constructed using 15 ng of total RNA. RNA was amplified using the WT-Ovation Pico RNA Amplification System and WT-Ovation Exon Module (NuGEN Technologies). Double-stranded cDNA was fragmented using NEBNext® dsDNA Fragmentase® (New England Biolabs) and Illumina-compatible libraries were made either by following the methods of Kumar et al. (1) for the globular, heart and cotyledon stages, or using Illumina TruSeq DNA Sample prep kit for the early maturation stage, according to manufacturer's instructions. For all samples, fifteen cycles of PCR enrichment steps were performed using the Pfu Turbo Cx hotstart DNA polymerase (Agilent).

Nuclei Isolation and Single Nucleus Sequencing. Nuclei were isolated following a modication of the procedures of Lu et al. (2). Approximately 50 cotyledon-stage seeds were chopped in 2 ml of pre-chilled lysis buffer (15 mM Tris pH 7.5, 20 mM NaCl, 80 mM KCl, 0.5 mM Spermine, 5 mM 2-ME, 0.05% Triton X-100, 80 U/ml RNase inhibitor (Roche, 3335399001)). The suspension was filtered through two layers of 40 μm and two layers of 20 μm nylon mesh, diluted with 3 volumes of cold wash buffer (20 mM MES pH 5.4, 0.4 M Mannitol, 20 mM KCl, 0.1% BSA, 80 U/ml RNase inhibitor) and pelleted at 4°C for 5 minutes at 400g in a swinging bucket rotor. Nuclei were resuspended in 1 ml of cold wash buffer, stained with DAPI and concentration was estimated using a hemocytometer. Nuclei were pelleted again and resuspended to a target concentration of 3500 nuclei/μl in 1X Nuclei Buffer (10X Genomics) containing 1 mM DTT and 1 U/μl RNase inhibitor. The nuclei suspension was filtered using a Flowmi™ Tip Strainer (Bel-Art) before proceeding with 10X Genomics Single-Cell Multiome ATAC + Gene Expression v1 Assay. Libraries were prepared at the UC Davis DNA Technologies and Expression Analysis Core Laboratory, according to manufacturer's instructions. Libraries were sequenced on a NovaSeq 6000 sequencing system.

Data Analysis.

Sample hierarchical clustering and PCA analysis. We identified quantitatively varying mRNAs using the EdgeR ANOVA-like test (FDR < 0.05) and selected the 15,000 mRNAs

with the highest SD to perform hierachical clustering and bootstrapping, and PCA analysis using Z-score normalized values as described in Belmonte et al. (3).

Weighted gene co-expression network analysis (WGCNA). We used the R package WGCNA to identify modules of co-expressed genes in the LCM dataset (4, 5). Abundant (> 1 cpm in ≥ 3 samples) and variable (50% highest CV) mRNAs were normalized using a variance-stabilizing transformation (DESeq2 v1.6.3)(6). A weighted network was constructed by transforming the mRNA-wise correlation matrix (biweight midcorrelation) into a signed hybrid adjacency matrix using a soft power threshold, then into a topological overlap matrix. Hierarchical clustering was used to identify modules of highly correlated mRNAs. The weighted a verage expression profile (module eigengene, ME) was used to merge similar modules (> 0.75). New MEs and eigengene-based connectivity (kME) were calculated for the merged modules, and mRNAs were assigned to each module using a fuzzy kME threshold of 0.75

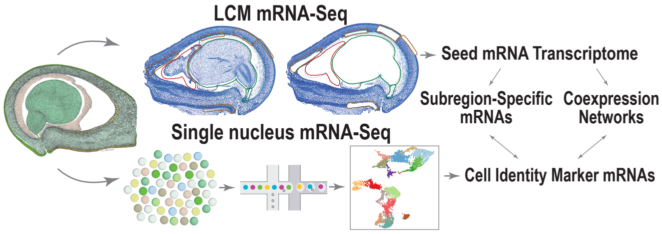

Figure S1. Strategies used to define gene networks operating in regions, subregions and cell types of developing soybean seeds.
LCM-mRNA Seq and snRNA Seq experiments were conducted to define subregion-specific mRNAs, cell identity marker snRNAs, and gene coexpression networks.

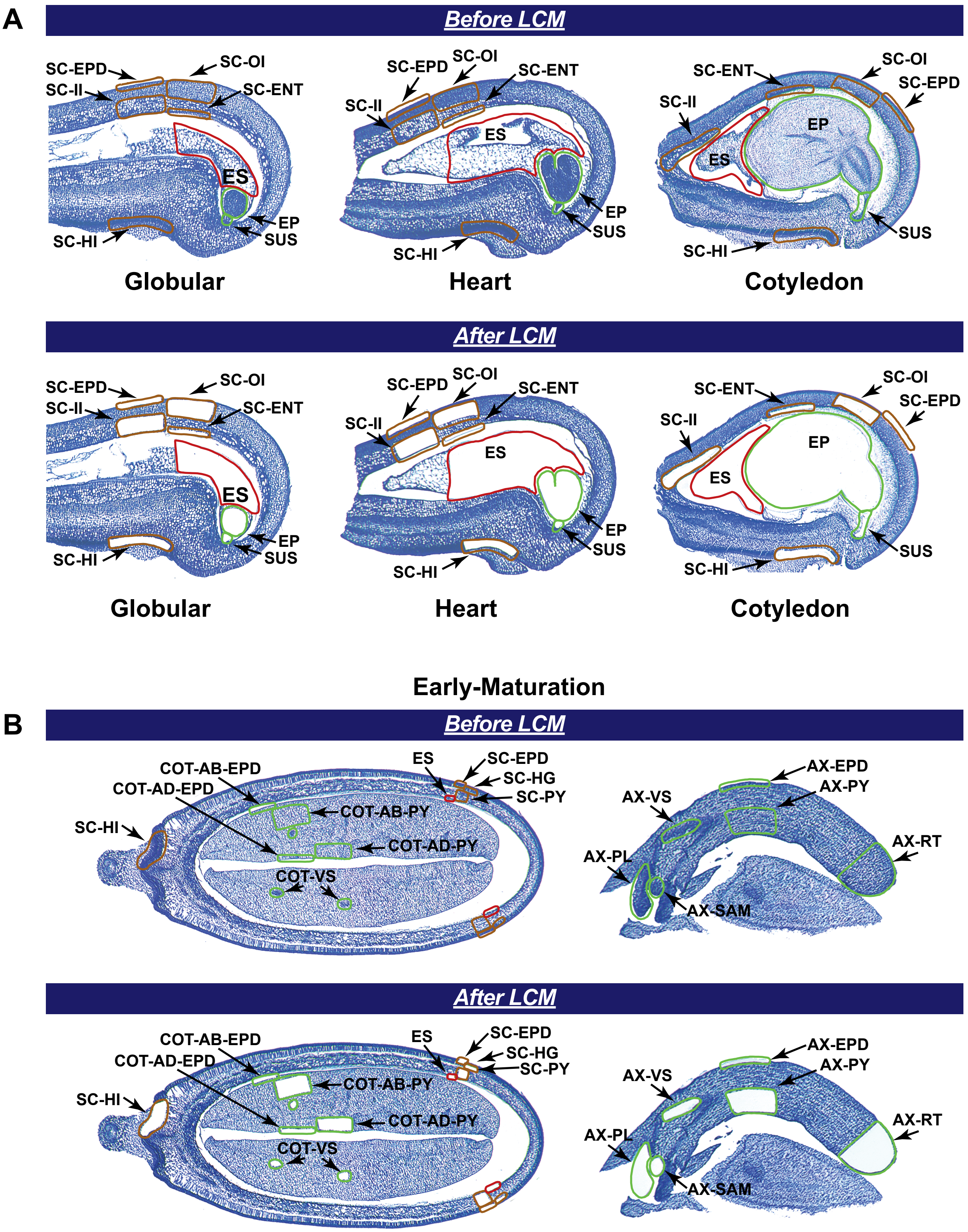

Figure S2. Isolation of soybean seed regions, subregions and tissues for mRNA profiling using LCM. *(A)* Longitudinal seed sections at the glob, hrt, and cot stages with different subregions outlined, before (top) and after (bottom) LCM. *(B)* Transverse seed section (left) and longitudinal embryo axis section (right) with em stage subregions outlined before (top) and after (bottom) LCM. Embryo, endosperm, and seed coat subregions are outlined in green, red, and brown, respectively.

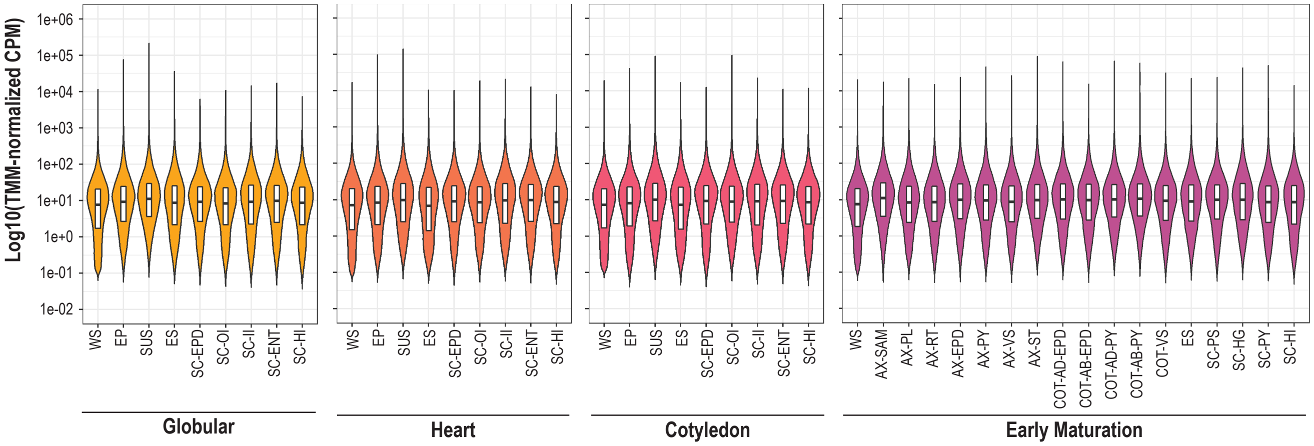

Figure S3. mRNA prevalence distribution in seed subregions.
Violin plot showing mRNA prevalences in seed subregions at the indicated stages of development.

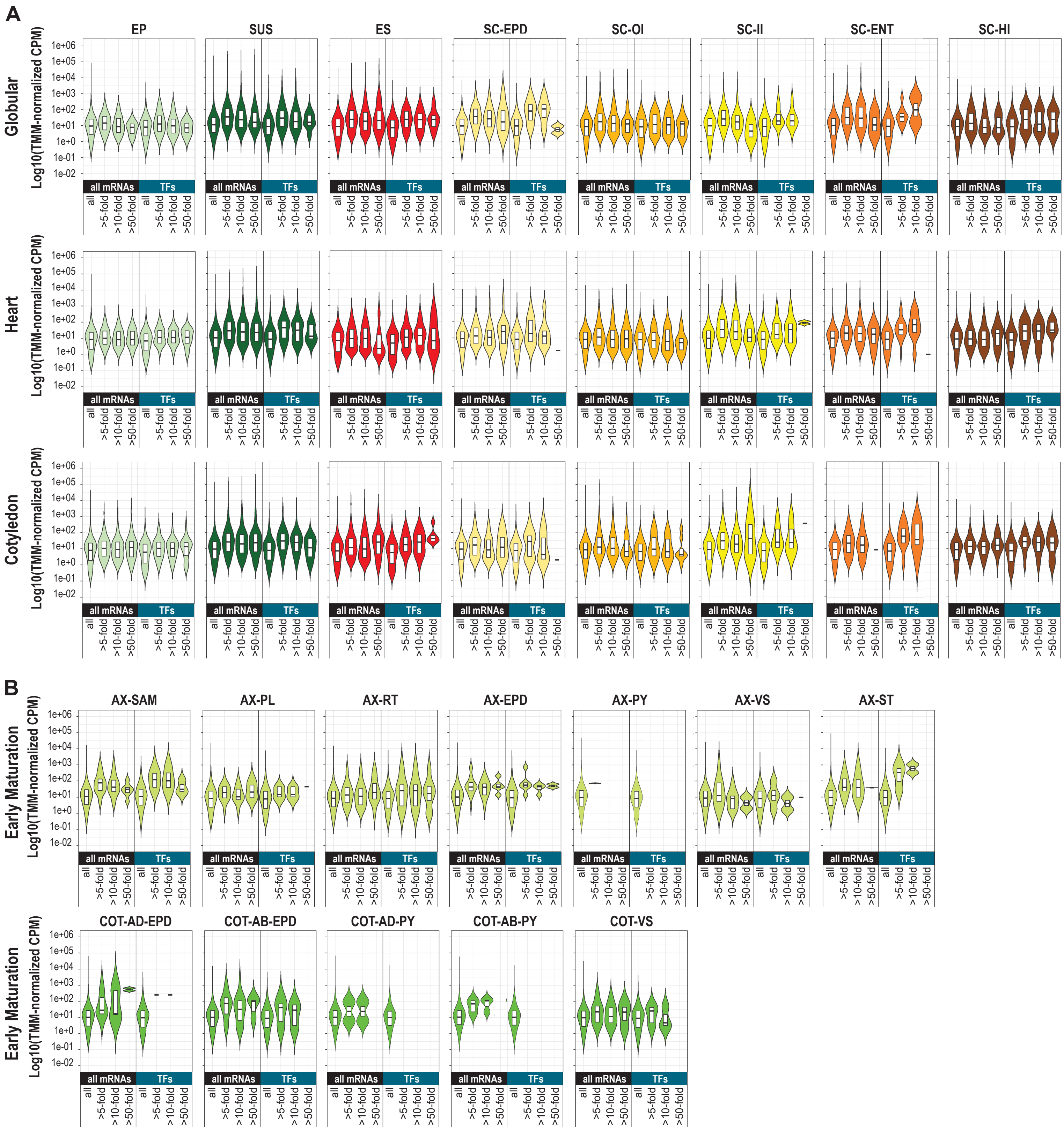

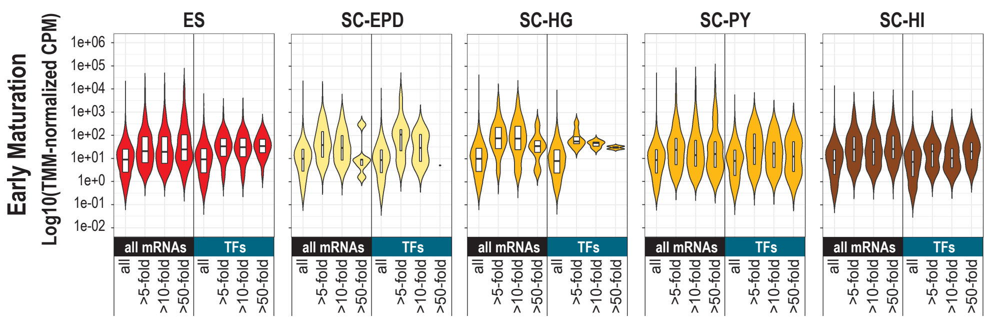

Figure S4. mRNA prevalence of enriched mRNAs in seed subregions during development.
Violin plots depict the distribution of all mRNAs (black bar, left) and TF mRNAs (teal bar, right) and those enriched in subregions at > 5-, > 10- and > 50-fold level at the glob, hrt and cot stages *(A)* and em stage *(B)*.

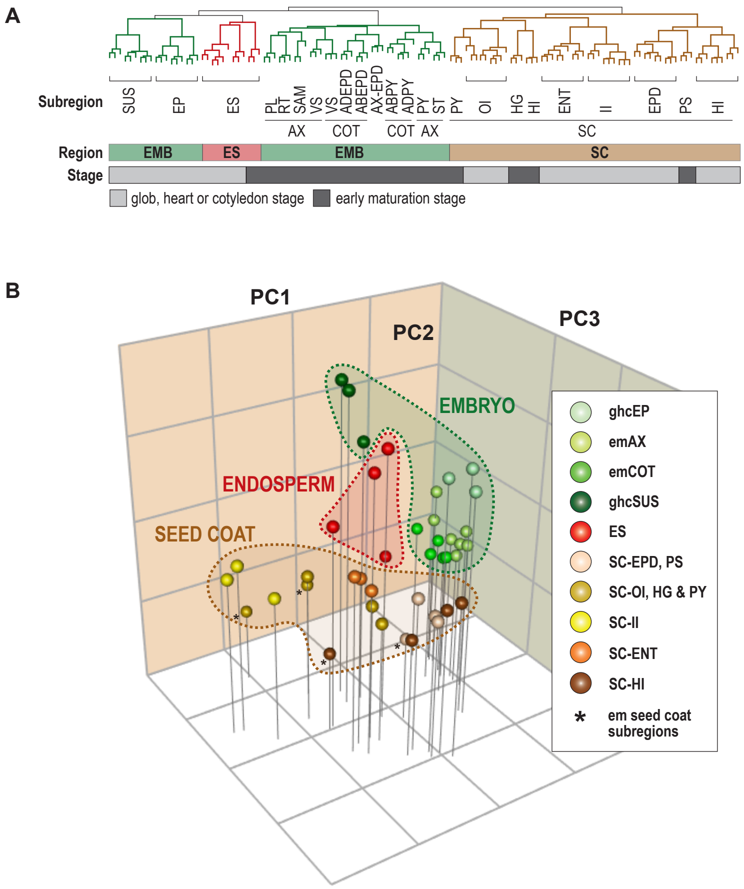

Figure S5. Biological relationships between seed subregions throughout development. *(A)* Hierarchical clustering of 41 distinct seed subregions throughout development. Bootstrap values of 111 of 121 branches was greater than 90, and 117 were over 70. Subregions, regions and developmental stages corresponding to each cluster are indicated. *(B)* Principal component analysis of the 41 LCM subregions, with principal components 1, 2, and 3 shown. Circles represent the seed subregions and stages. Embryo, endosperm, and seed coat subregions are outlined in green, red, and brown, respectively, and early maturation seed coat subregions are marked with an asterisk. Abbreviations are as in Fig. 1, except ghc, globular, heart, cotyledon stages.

Globular Stage

Embryo

EMBRYO PROPER

GRF6	Glyma.U028700
GRF1	Glyma.17G232600
CUC2	Glyma.13G274300
STM	Glyma.09G007500
AIL6	Glyma.01G022500
WRI1	Glyma.08G227700
NF-YA3	Glyma.09G068400
STM	Glyma.15G111900
KNAT6	Glyma.08G284300
SAP	Glyma.02G304600

SUSPENSOR

WOX9	Glyma.11G210800
ERF019-like	Glyma.11G014800
ARF19	Glyma.01G002100
LEC1-1	Glyma.07G268100
HAT22	Glyma.15G168800
bHLH113-like	Glyma.20G130200
WIP5	Glyma.12G057000
WOX9	Glyma.13G172700
STO	Glyma.15G029500
NIN-like	Glyma.15G027900

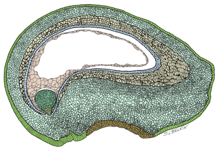

ENDOSPERM

AGL6	Glyma.09G266400
NAC2	Glyma.12G221400
NAC083	Glyma.05G202300
BLH1	Glyma.U009200
WRKY2	Glyma.18G242000
NAC072	Glyma.06G248900
NAC2	Glyma.06G249100
AGL62	Glyma.10G085700
EIN3	Glyma.06G314000
C2H2 ZF TF	Glyma.03G033600

Seed coat

EPIDERMIS

TTG2	Glyma.19G177400
TCP3	Glyma.06G232300
ETC1/MYB172	Glyma.13G307300
TINY2	Glyma.08G035000
TCP3	Glyma.12G228300
ANT	Glyma.U037700
AtGRF5	Glyma.13G109500
GL2	Glyma.13G357100
MYB5	Glyma.05G061900
SPL13	Glyma.06G168600

OUTER INTEGUMENT

IDD4	Glyma.07G158200
WOX1	Glyma.20G017600
SPL4	Glyma.U025800
SPL4	Glyma.06G238100
bZIP17	Glyma.17G253200
IDD5	Glyma.10G280000
HB24	Glyma.11G068800
Trihelix TF	Glyma.15G234100
HAT2	Glyma.01G207300
PIF3	Glyma.19G224700

INNER INTEGUMENT

NAC087	Glyma.19G109100
OZF1	Glyma.06G050300
NAC100	Glyma.17G101500
IDD4	Glyma.07G158000
UIF1-like	Glyma.07G178500
NAC074	Glyma.10G037700
DOF11	Glyma.13G329000
ARR1	Glyma.20G035300
NAC025	Glyma.12G171600
C2H2 ZF TF	Glyma.11G142500

ENDOTHELIUM

JKD	Glyma.13G139000
SOM	Glyma.12G116900
FUF1	Glyma.11G014200
DOF TF	Glyma.13G177500
WRKY32	Glyma.01G056800
DOF1	Glyma.07G012100
MYB84	Glyma.05G234600
MYB105	Glyma.01G049600
MYB56	Glyma.04G205100
LBD12	Glyma.18G024400

HILUM

DOF21	Glyma.04G233300	SUP	Glyma.12G230200
BEL1	Glyma.11G022200	bZIP65	Glyma.03G142400
KAN2	Glyma.09G207300	PHB	Glyma.15G129700
KAN2	Glyma.01G014800	HAT14	Glyma.02G171300
DOF28	Glyma.06G131500	LRP1	Glyma.07G230400

Heart Stage

Embryo

EMBRYO PROPER

GRF1	Glyma.17G232600
ANT	Glyma.14G089200
PHAN	Glyma.07G132400
AP2/B3-like TF	Glyma.06G038900
CUC2	Glyma.13G274300
ANT	Glyma.04G047900
STM	Glyma.09G007500
CUC2	Glyma.12G226500
GRF4	Glyma.19G192700
GRAS TF	Glyma.13G081700

SUSPENSOR

WOX9	Glyma.11G210800
WOX9	Glyma.10G069700
ERF019-like	Glyma.11G014800
ARF16	Glyma.10G210600
HAT22	Glyma.15G168800
HAT5	Glyma.17G096700
bHLH113-like	Glyma.20G130200
WRKY11	Glyma.04G076200
CRF4	Glyma.03G094700
NAC073	Glyma.13G234700

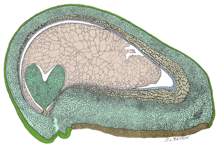

ENDOSPERM

AGL62	Glyma.20G136600
MYB98	Glyma.19G062000
MIF2	Glyma.09G238600
ARF9	Glyma.03G208800
ATML1	Glyma.10G251300
ATML1	Glyma.20G142200
AIL7	Glyma.08G279000
MIF2	Glyma.09G238500
MYB98	Glyma.16G072700
HDG11	Glyma.09G025800

Seed coat

EPIDERMIS

TCP3	Glyma.06G232300
TINY2	Glyma.08G035000
TCP2	Glyma.08G097900
TCP3	Glyma.12G228300
SPL13	Glyma.06G168600
GRF5	Glyma.13G109500
MYB16	Glyma.13G061900
MYB17	Glyma.09G261600
MYB5	Glyma.05G061900
bZIP50	Glyma.12G121000

OUTER INTEGUMENT

MYB138	Glyma.02G026300
SPL4	Glyma.U025800
IDD4	Glyma.20G184000
WOX1	Glyma.20G017600
WOX1	Glyma.07G219400
bZIP44	Glyma.14G071400
bZIP44	Glyma.17G253200
SPL4	Glyma.06G238100
G2-like TF	Glyma.17G205700
HB24	Glyma.11G068800

INNER INTEGUMENT

ANAC087	Glyma.19G109100
Trihelix TF	Glyma.03G246000
OZF1	Glyma.06G050300
DOF11	Glyma.13G329000
LBD4	Glyma.08G169000
HSFA6B	Glyma.03G157300
SCL3	Glyma.01G226600
ARR1-like	Glyma.20G035300
C2H2 ZF TF	Glyma.11G192600
NAC074	Glyma.10G037700

ENDOTHELIUM

JKD	Glyma.10G051500
JKD	Glyma.13G139000
SOM	Glyma.12G116900
HB17	Glyma.07G218000
AGL6	Glyma.07G081300
TT16	Glyma.20G001900
WRKY49	Glyma.07G133700
DOF TF	Glyma.13G177500
SOM	Glyma.12G205700
TT1	Glyma.14G174900

HILUM

SRS7	Glyma.16G132100	SUP	Glyma.12G230200
SHI	Glyma.01G170700	bZIP65	Glyma.19G145300
DAG1	Glyma.04G233300	DOF28	Glyma.06G131500
IND1	Glyma.05G190600	bZIP65	Glyma.03G142400
KAN2	Glyma.01G014800	bZIP65	Glyma.10G092100

Cotyledon Stage

Embryo

EMBRYO PROPER

YAB1	Glyma.03G029800
YAB1	Glyma.01G137200
WRI1	Glyma.08G227700
AIL1	Glyma.07G038200
HB22	Glyma.09G170500
BEH4	Glyma.12G231500
MYC67	Glyma.16G017700
bHLH96-like	Glyma.08G271000
HB22	Glyma.06G095200
HB22	Glyma.04G093300

SUSPENSOR

WOX9	Glyma.10G069700
WOX9	Glyma.11G210800
ERF019-like	Glyma.11G014800
PLT2	Glyma.12G056300
LEC1-4	Glyma.20G000600
BBM	Glyma.09G248200
ARF19,	Glyma.07G130400
PLT2	Glyma.11G131900
ARF19	Glyma.01G002100
MYB30	Glyma.19G022100

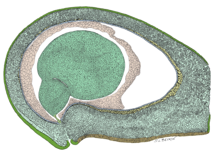

ENDOSPERM

RL1	Glyma.04G031700
MIF2	Glyma.09G238600
HDG11	Glyma.09G025800
MYB98	Glyma.19G062000
LBD41	Glyma.08G261900
MIF1	Glyma.18G257200
MYB98	Glyma.16G072700
HB22	Glyma.12G168600
MIF2	Glyma.09G238700
GATA9	Glyma.02G073900

Seed coat

EPIDERMIS

TINY2	Glyma.08G035000
MYB5	Glyma.05G061900
SPL13	Glyma.06G168600
MYB17	Glyma.09G261600
bHLH25-like	Glyma.07G027400
SPL13	Glyma.04G197100
MYB5	Glyma.19G257400
AGL4	Glyma.18G273500
MYB4	Glyma.03G258700
MYB3	Glyma.07G216000

OUTER INTEGUMENT

bZIP25	Glyma.04G010300
MYB138	Glyma.02G026300
bZIP124	Glyma.12G040600
bZIP62	Glyma.06G079800
bZIP17	Glyma.14G071400
bIP17	Glyma.17G253200
WOX1	Glyma.07G219400
AG	Glyma.08G120600
ABS5	Glyma.14G181500
BHLH32	Glyma.02G213800

INNER INTEGUMENT

Trihelix TF	Glyma.03G246000
ANAC087	Glyma.19G109100
OZF1	Glyma.06G050300
Trihelix TF	Glyma.19G243500
NAC074	Glyma.10G037700
ANAC087	Glyma.16G042900
ANAC087	Glyma.16G152100
UIF1-like	Glyma.07G178500
DOF11	Glyma.13G329000
C2H2 ZF TF	Glyma.11G192600

ENDOTHELIUM

JKD	Glyma.10G051500
JKD	Glyma.13G139000
HB17	Glyma.07G218000
AGL6	Glyma.03G019400
TT16	Glyma.20G001900
WRKY49	Glyma.07G133700
TCP14	Glyma.06G193000
TT16	Glyma.09G283800
BLH1	Glyma.12G188800
COG1	Glyma.05G158200

HILUM

IND1	Glyma.05G190600	bZIP65	Glyma.02G176800
DAG1	Glyma.04G233300	SRS7	Glyma.06G027500
SRS7	Glyma.16G132100	bZIP65	Glyma.10G092100
VDOF1	Glyma.06G131500	SUP	Glyma.10G034600
SUP	Glyma.12G230200	LRP1	Glyma.11G155400

Early Maturation Stage

Embryo Axis

SHOOT APICAL MERISTEM

BLH8	Glyma.12G077800
ERF TF	Glyma.07G027000
BLH8	Glyma.11G196300
DAG1	Glyma.04G233300
NA	Glyma.08G215700
bZIP46-like	Glyma.02G045500
SPL13	Glyma.05G204100
ERF TF	Glyma.01G025400
ERF TF	Glyma.18G144700
HB30	*Glyma.18G259300*

EPIDERMIS

WOX9	Glyma.10G069700
WOX8	Glyma.07G203000
BBM	Glyma.10G171400
IDD7	Glyma.10G020200
MYB5	Glyma.01G207600
ZFP2	Glyma.17G064700
LEC1-2	*Glyma.17G005600*
GRF5	*Glyma.17G050200*
HB30	*Glyma.09G238400*
AIL5	*Glyma.13G096900*

PLUMULE

TCP1	Glyma.08G256400
LFY, LFY3	Glyma.04G202000
PTL	Glyma.20G188100
NA	Glyma.07G158600
TCP1	Glyma.18G280700
YAB1	*Glyma.03G029800*
GRF5	*Glyma.17G050200*
GRF1	*Glyma.U028600*
AIL1	*Glyma.16G007400*
AIL1	*Glyma.07G038200*

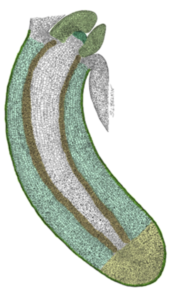

ROOT TIP

PLT2	Glyma.11G131900
PLT2	Glyma.12G056300
SMB	Glyma.08G161300
SMB	Glyma.15G266500
FEZ	Glyma.08G301100
AGL21	Glyma.09G201700
FEZ	Glyma.02G107000
NAC094	Glyma.19G021900
LBD31	Glyma.01G143800
WOX5	Glyma.02G254800

ENDOSPERM

C2H2 TF	Glyma.06G033300	MYB98	Glyma.19G062000
HDG11	Glyma.09G025800	LBD41	Glyma.08G261900
NAC075	Glyma.07G126500	NAC073	Glyma.07G201800
ERF TF	Glyma.17G254600	MYB98	Glyma.05G005400
ARF9	Glyma.03G208800	MYB98	Glyma.16G072700

Embryo Cotyledon

PARENCHYMA

HSFB4	*Glyma.02G278400*
B3 TF	*Glyma.14G079100*
BBM	*Glyma.09G248200*
AIL5	*Glyma.13G096900*
KNAT1	*Glyma.U039200*
ABI5	*Glyma.10G071700*
GRF1	*Glyma.17G232600*
GRF5	*Glyma.17G050200*
bHLH70-like	*Glyma.07G049100*
BBM	*Glyma.18G244600*

ADAXIAL EPIDERMIS

ASL1	Glyma.01G183900
LEC1-2	*Glyma.17G005600*
TCP3	*Glyma.06G232300*
GRF1	*Glyma.U028600*
TCP3	*Glyma.12G158900*
ICE1	*Glyma.04G200500*
AIL5	*Glyma.13G096900*
AIL1	*Glyma.07G038200*
GRF1	*Glyma.17G232600*
SPCH	*Glyma.06G125500*

VASCULATURE

WRKY7	Glyma.17G239200
NA	Glyma.15G076000
LBD39	Glyma.17G236300
TT2	Glyma.16G007200
bHLH106-like	*Glyma.19G128900*
KNAT1	*Glyma.U039200*
GRF5	*Glyma.17G050200*
BBM	*Glyma.09G248200*
BBM	*Glyma.18G244600*
GRF1	*Glyma.17G232600*

VASCULATURE

PFA4	Glyma.13G249800
MYB59	Glyma.12G066000
LBD21	Glyma.18G025600
LBD4	Glyma.09G021600
PI	Glyma.06G117600
LEC1-2	*Glyma.17G005600*
HB22	*Glyma.09G170500*
BEH4	*Glyma.13G266500*
bHLH106-like	*Glyma.19G128900*
BEH4	*Glyma.12G231500*

ABAXIAL EPIDERMIS

KNAT3	Glyma.15G202200
WOX1	Glyma.18G179300
TCP5	Glyma.17G079900
TCP5	Glyma.06G204300
LEC1-3	Glyma.03G080700
MYB113	Glyma.07G141100
AIL5	*Glyma.13G096900*
LEC1-2	*Glyma.17G005600*
TCP3	*Glyma.12G158900*
YAB1	*Glyma.03G029800*

STELE

IDD12	Glyma.10G029700
IDD12	Glyma.02G144400
UNE10	Glyma.03G034000
GRF1	*Glyma.U028600*
B3 TF	*Glyma.14G079100*
KNAT1	*Glyma.U039200*
B3 TF	*Glyma.17G246000*
STM	*Glyma.14G047000*
ARF9	*Glyma.16G023600*
ABI5	*Glyma.10G071700*

ADAXIAL PARENCHYMA

LEC1-2	*Glyma.17G005600*
GRF1	*Glyma.U028600*
TCP3	*Glyma.12G158900*
GRF5	*Glyma.16G007600*
GRF1	*Glyma.17G232700*
WRI1	*Glyma.08G227700*
GRF1	*Glyma.17G232600*
GRF5	*Glyma.07G038400*
HB22	*Glyma.04G093300*
GRF7	*Glyma.04G230600*

ABAXIAL PARENCHYMA

LEC1-2	*Glyma.17G005600*	*HB22*	*Glyma.04G093300*
GRF1	*Glyma.U028600*	*WRI1*	*Glyma.08G227700*
GRF5	*Glyma.16G007600*	*ABI5*	*Glyma.13G153200*
ARF9	*Glyma.16G023600*	*GRF1*	*Glyma.17G232600*
YAB1	*Glyma.03G029800*	*GRF5*	*Glyma.07G038400*

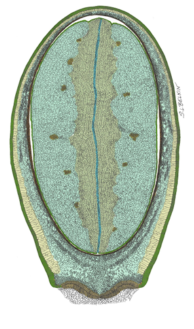

Seed Coat

PALISADE

ETC1	Glyma.13G307300
GL2	Glyma.13G357100
AGL9	Glyma.05G148800
SPL13	Glyma.06G168600
MYB5	Glyma.17G143600
AP2	Glyma.05G091200
MYB5	Glyma.05G061900
MYB4	Glyma.03G258700
MYB3	Glyma.07G216000
LBD11	Glyma.13G328500

HOURGLASS

MIF2	Glyma.13G063100
MYB86-like	Glyma.05G040700
ERF4	Glyma.13G274100
MYB86-like	Glyma.17G085600
PRE5	Glyma.13G063800
REM19	Glyma.12G048700
ERF TF	Glyma.03G258200
ERF TF	Glyma.19G256800
CO-like	*Glyma.08G041100*
MYB61	*Glyma.20G090700*

PARENCHYMA

bZIP124	Glyma.12G040600
HAT22	Glyma.17G157600
MYB138	Glyma.02G026300
bZIP17	Glyma.14G071400
WOX1	Glyma.07G219400
DREB2	Glyma.08G137600
MIF2	Glyma.19G022200
NF-YB3	Glyma.08G141000
HB40	Glyma.11G064800
HB40	Glyma.01G177500

HILUM

IND1	Glyma.05G190600	BEL1	Glyma.05G210300
SUP	Glyma.10G034600	WRKY28	Glyma.14G028900
NA	Glyma.03G033600	MYBJ2	Glyma.05G027000
MYB55	Glyma.10G139000	SUP	Glyma.13G269400
SUP	Glyma.13G120900	IND1	Glyma.06G152300

Figure S6. Specialization of TF mRNAs in seed subregions throughout development.
Ten most prevalent subregion-specific or regional-specific (italicized) transcription factors in each subregion, at the globular, heart, cotyledon and early maturation stage are listed.

A

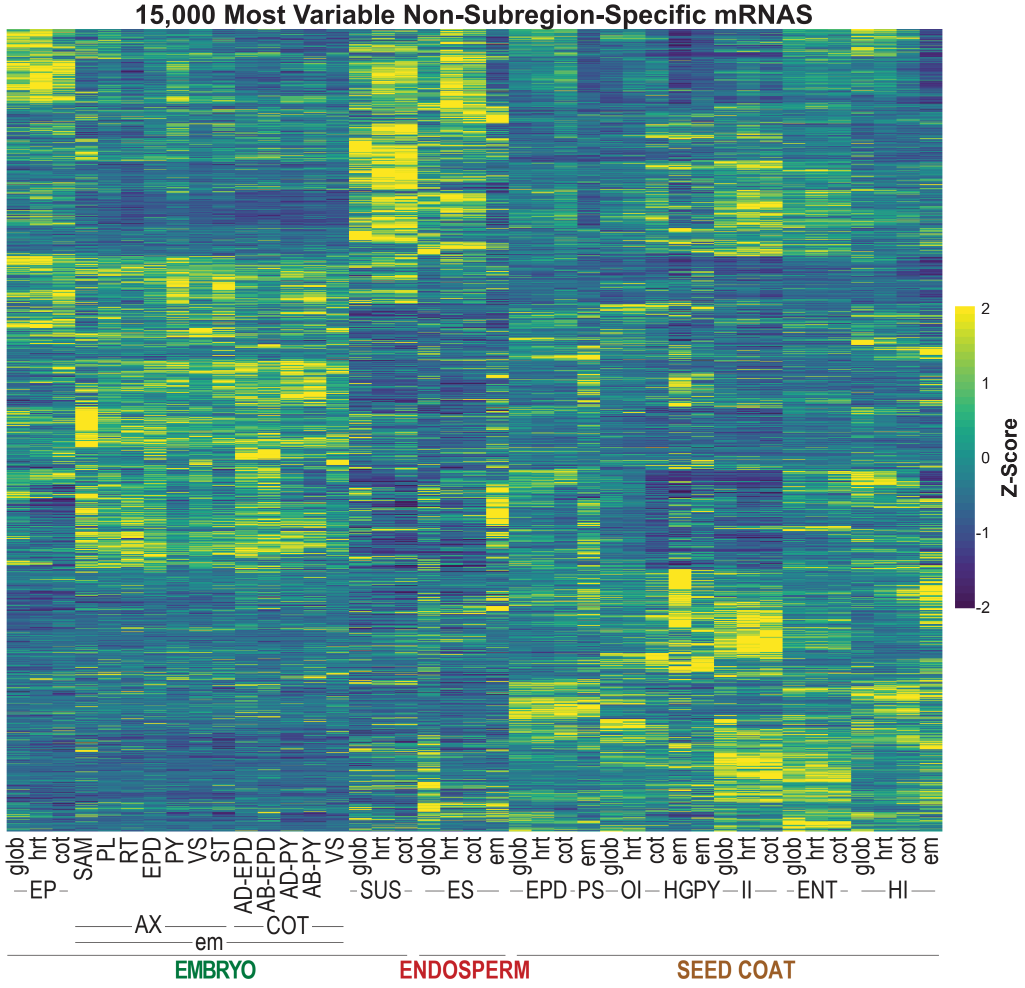

B

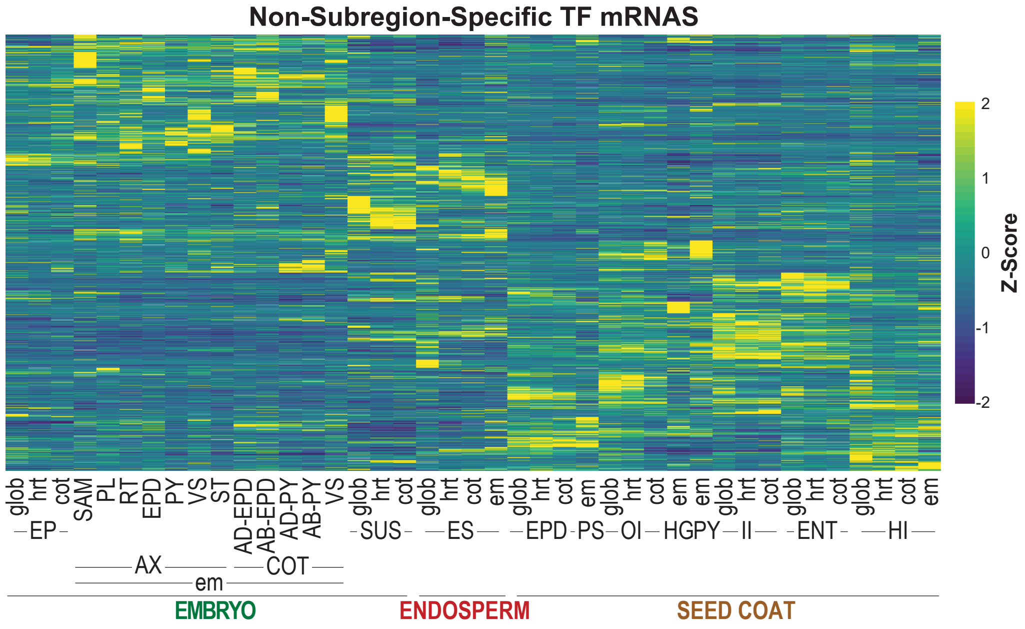

Figure S7. mRNAs that are not subregion specific accumulate primarily in a specific subregion. Hierarchical clustering of the 15,000 most varying (FDR < 0.05) non-subregion specific mRNAs (A) and non-subregion specific TF mRNAs (B) in different subregions and stages. Heatmap represents the z-score of mRNA levels across subregions and stages.

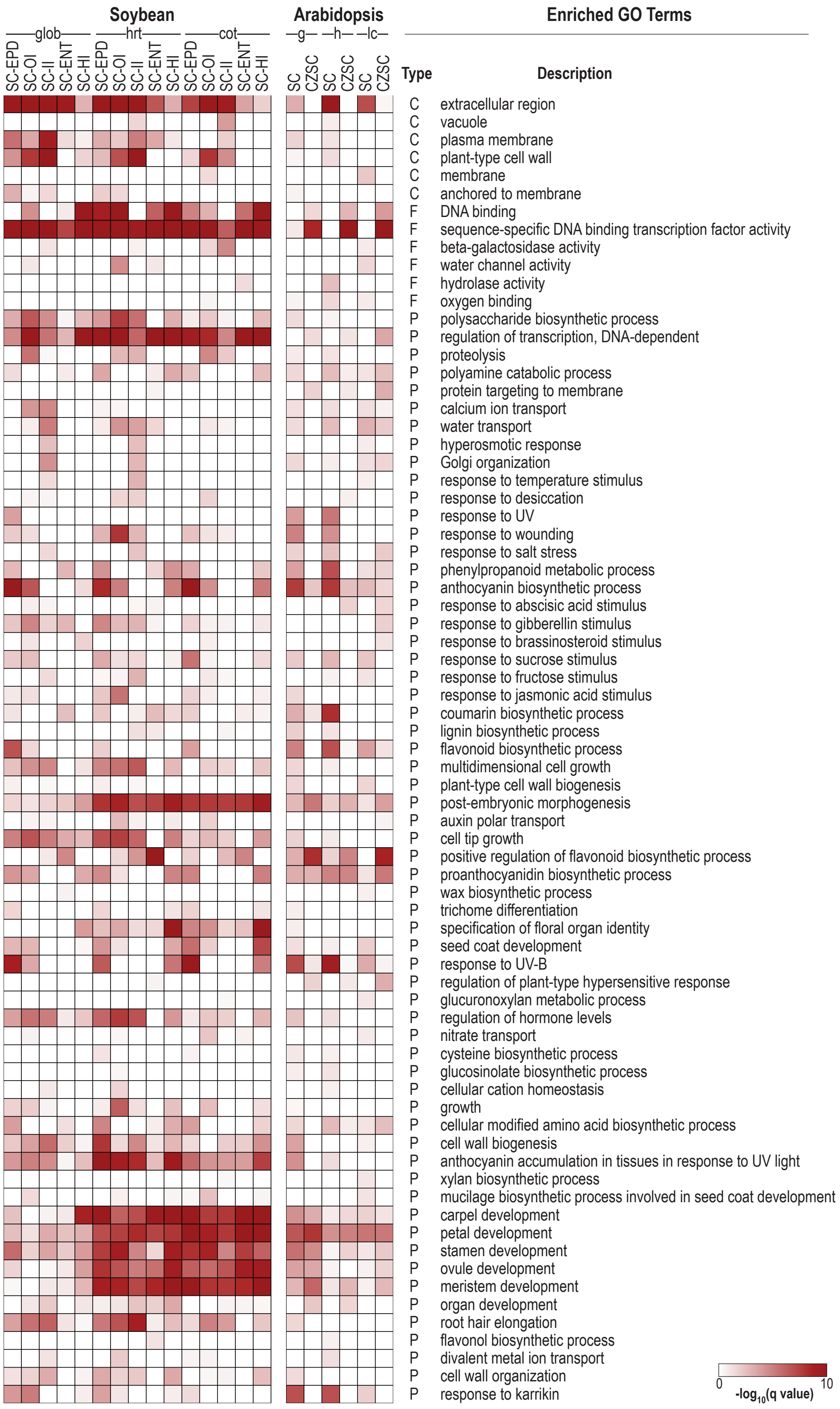

Figure S8. Conserved processes in soybean and Arabidopsis seed coat subregions.
Heatmap of the significance of enrichment for regional-specific GO terms in seed coat subregions of glob, hrt, and cot-stage seeds.

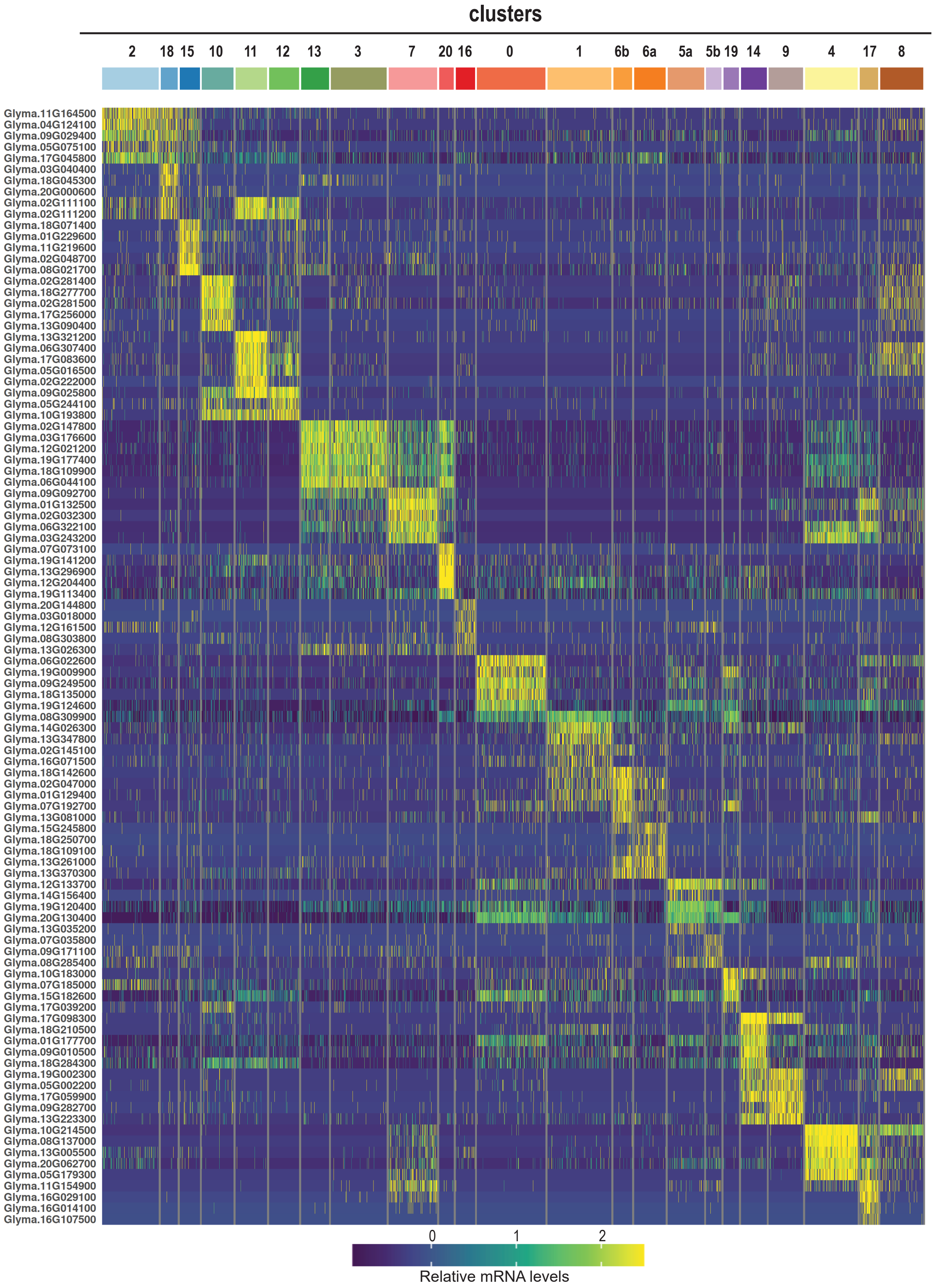

Figure S9. Relative levels of the five most prevalent marker snRNAs in each of the 23 clusters. Heatmap of the snRNA levels are shown.

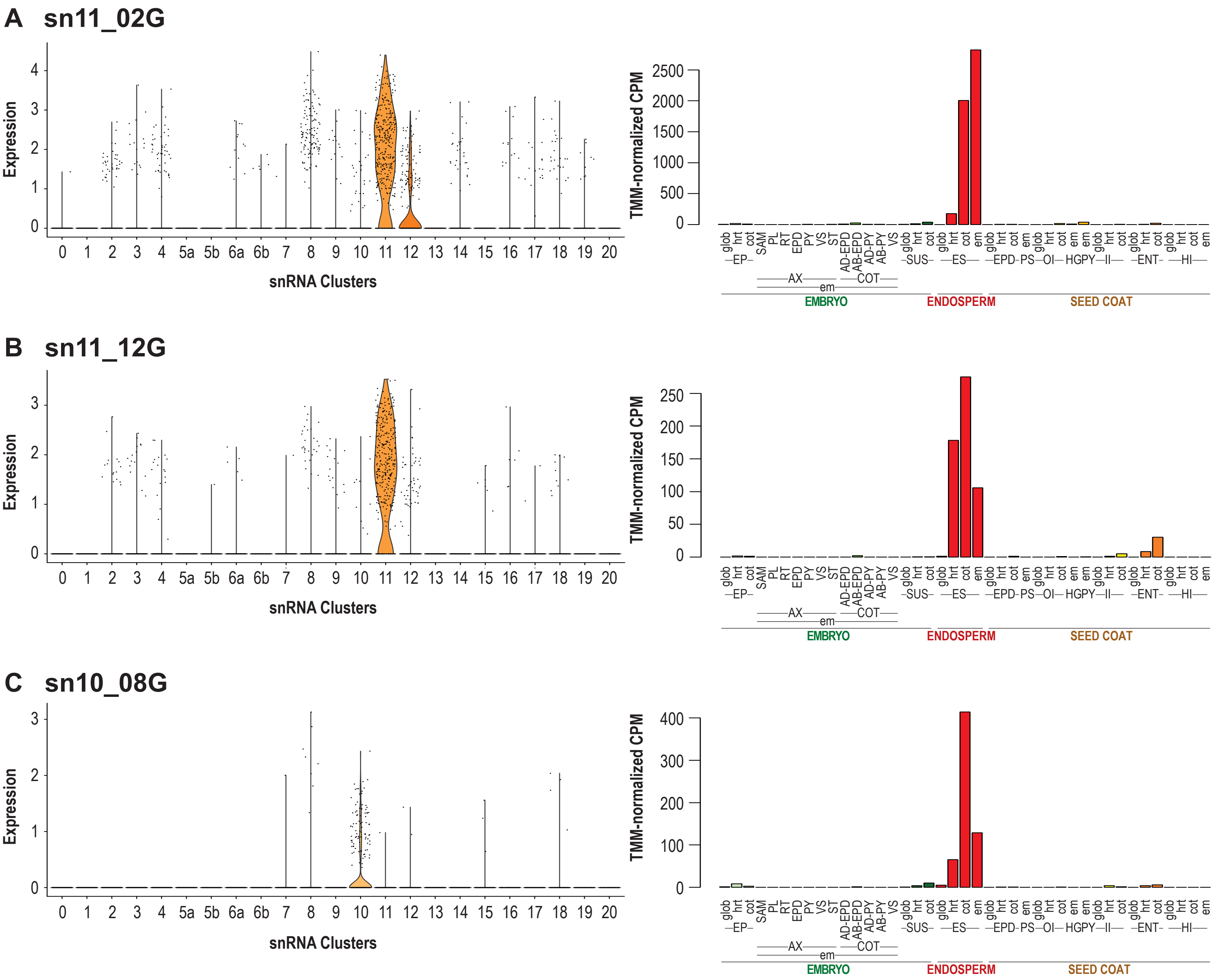

Figure S10. Abundance of HCR marker mRNAs.
Violin plot showing the distribution of the indicated snRNA levels in nuclei for each cluster *(left)* and of the mRNA in each subregion *(right)* for markers selected for HCR experiments: *(A)* sn11_02G, *(B)* sn11_12G, and *(C)* sn10_08G.

A

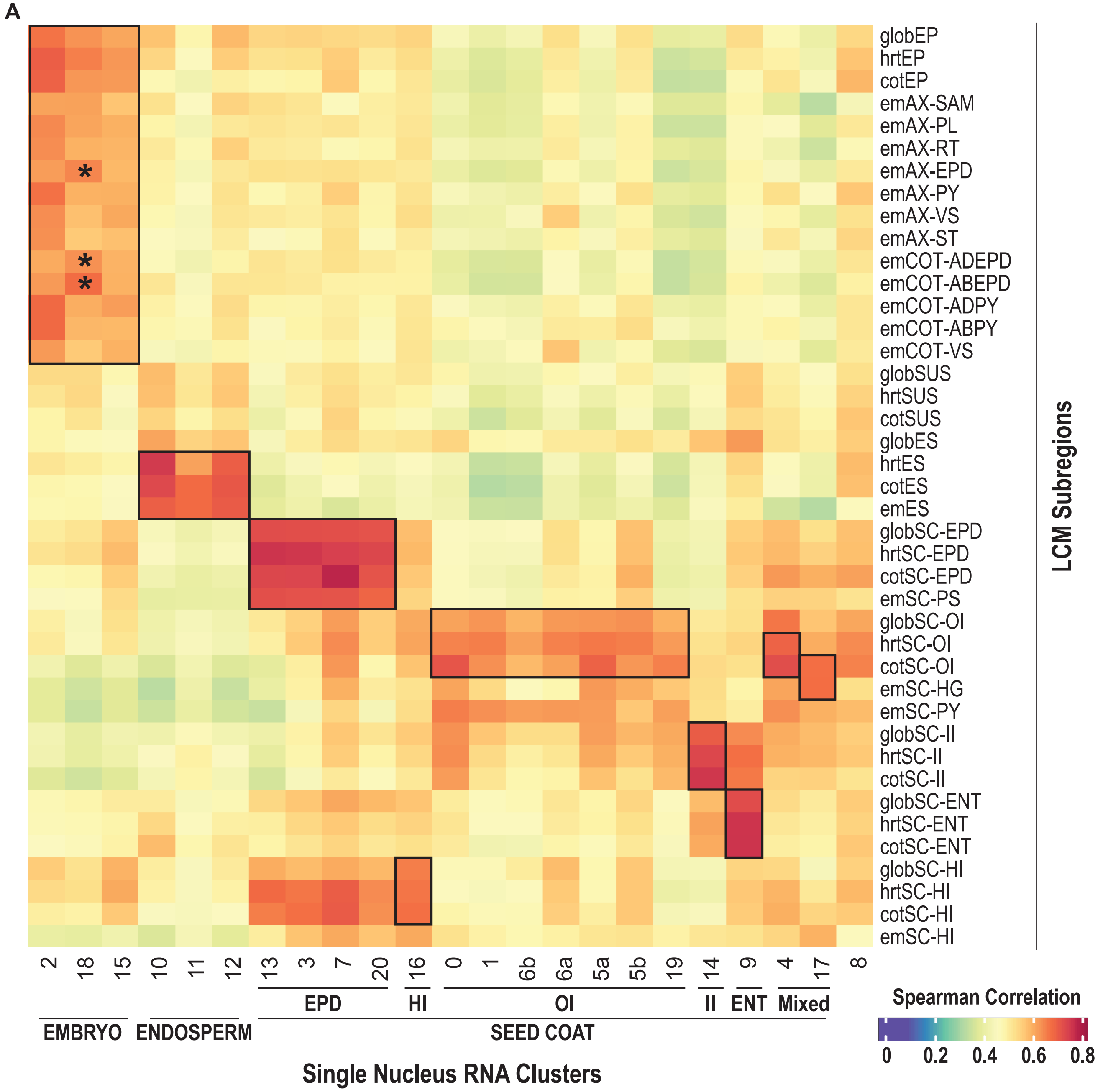

globEP
hrtEP
cotEP
emAX-SAM
emAX-PL
emAX-RT
emAX-EPD
emAX-PY
emAX-VS
emAX-ST
emCOT-ADEPD
emCOT-ABEPD
emCOT-ADPY
emCOT-ABPY
emCOT-VS
globSUS
hrtSUS
cotSUS
globES
hrtES
cotES
emES
globSC-EPD
hrtSC-EPD
cotSC-EPD
emSC-PS
globSC-OI
hrtSC-OI
cotSC-OI
emSC-HG
emSC-PY
globSC-II
hrtSC-II
cotSC-II
globSC-ENT
hrtSC-ENT
cotSC-ENT
globSC-HI
hrtSC-HI
cotSC-HI
emSC-HI
LCM Subregions
2 18 15 10 11 12 13 3 7 20 16 0 1 6b 6a 5a 5b 19 14 9 4 17 8
EPD
HI
OI
II
ENT
Mixed
EMBRYO
ENDOSPERM
SEED COAT
Single Nucleus RNA Clusters
Spearman Correlation
0
0.2
0.4
0.6
0.8

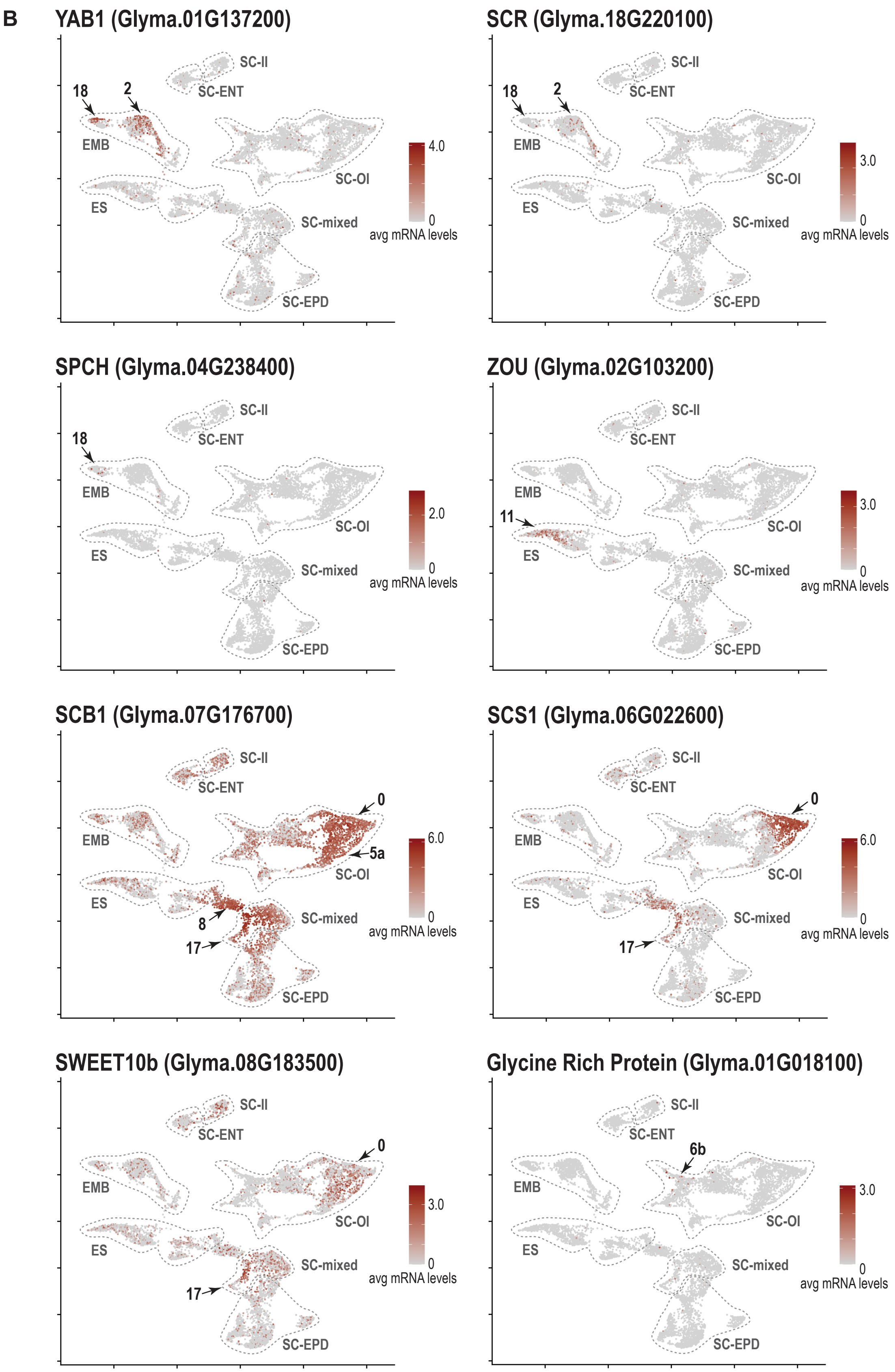

Figure S11. Validation of snRNA cluster assignments to seed subregions.
(A) Heatmap of the Spearman rank correlation between cluster average snRNA levels and LCM mRNA levels of the 15,000 most varying snRNAs across clusters. *(B)* Mapping of reference mRNAs listed in Fig. 6 to snRNA clusters. Heatmaps represent mRNA prevalence in UMAP representation of the snRNA clusters. snRNA clusters associated with subregions are outlined (dotted line), and clusters enriched for reference mRNAs are numbered.

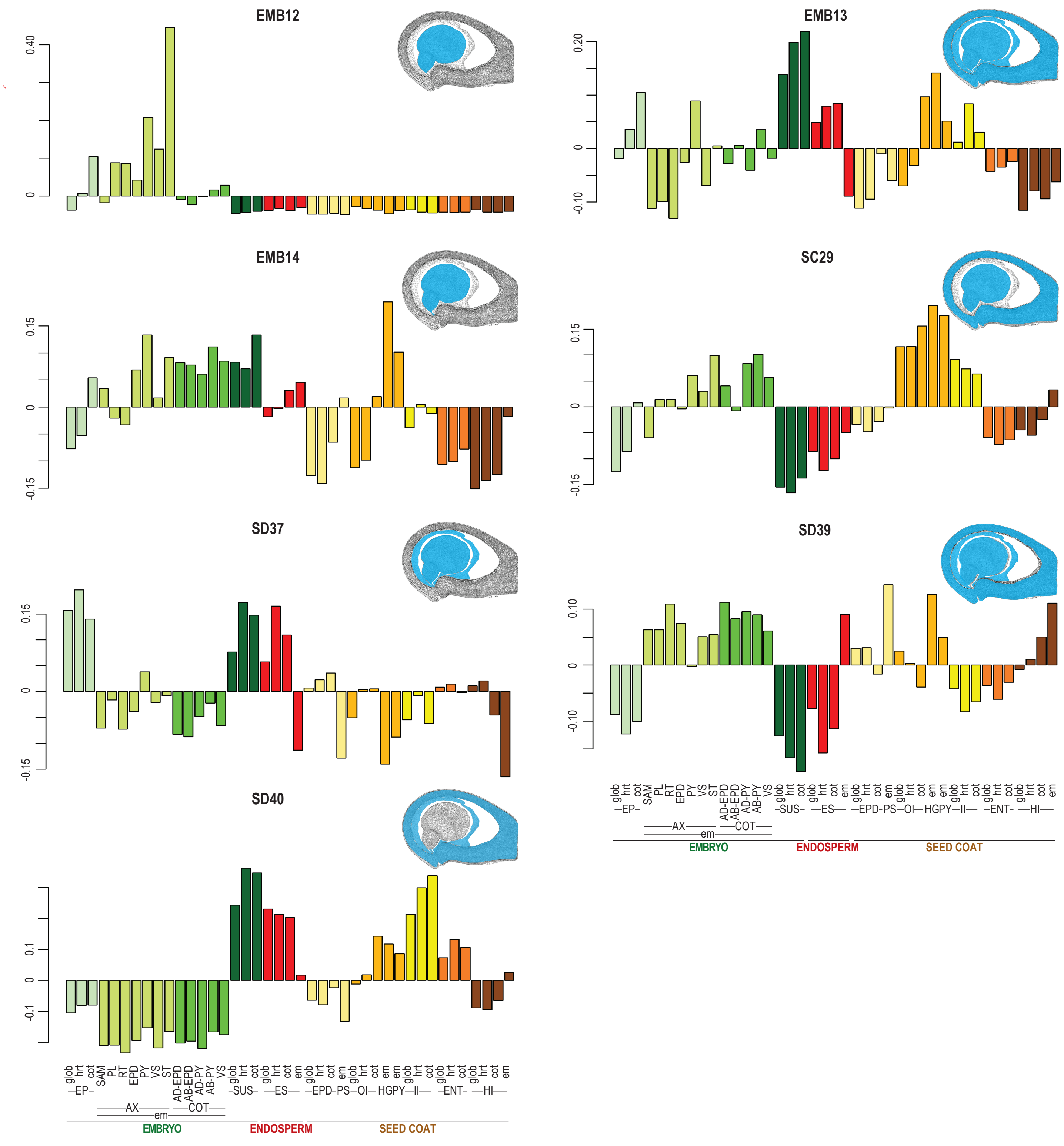

Figure S12. Seed coexpression networks.
Other WGCNA modules not presented in Figures 7 or 8.

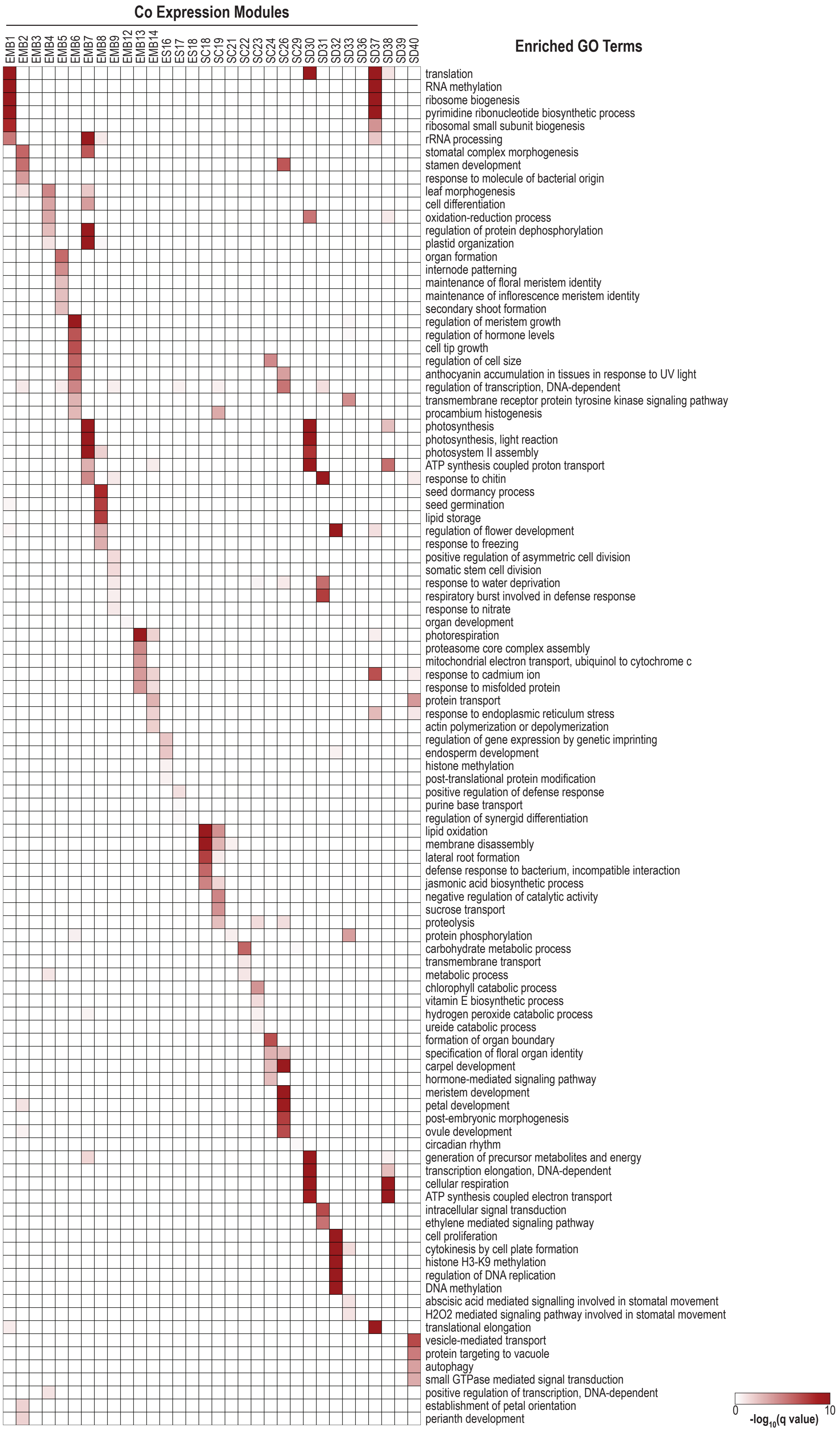

Figure S13. Gene coexpression networks are enriched for distinct biological processes. Heatmap of the significance of enrichment for the top five biological process GO terms for each of the non-epidermal WGCNA modules.

Table S1. Pairwise Pearson correlations.

Comparisons between biological replicates are highlighted.

	globEP-4	globEP-5	globEP-6	hrtEP-1	hrtEP-2	hrtEP-3	cotEP-1	cotEP-2	cotEP-3	emAX-SAM-2	emAX-SAM-3	emAX-SAM-4	emAX-PL-1	emAX-PL-2	emAX-PL-3	emAX-RT-1	emAX-RT-2	emAX-RT-3
globEP-4	1.000	0.998	0.992	0.992	0.993	0.988	0.902	0.828	0.914	0.340	0.330	0.202	0.418	0.450	0.484	0.233	0.257	0.248
globEP-5	0.998	1.000	0.990	0.987	0.991	0.989	0.912	0.842	0.922	0.359	0.349	0.217	0.432	0.471	0.503	0.252	0.282	0.270
globEP-6	0.992	0.990	1.000	0.977	0.980	0.978	0.898	0.841	0.915	0.374	0.372	0.225	0.455	0.489	0.516	0.283	0.308	0.300
hrtEP-1	0.992	0.987	0.977	1.000	0.994	0.991	0.905	0.828	0.919	0.316	0.302	0.183	0.399	0.427	0.465	0.202	0.220	0.215
hrtEP-2	0.993	0.991	0.980	0.994	1.000	0.994	0.899	0.824	0.913	0.314	0.303	0.179	0.402	0.437	0.471	0.214	0.242	0.230
hrtEP-3	0.988	0.989	0.978	0.991	0.994	1.000	0.921	0.857	0.933	0.351	0.339	0.203	0.430	0.468	0.501	0.241	0.271	0.259
cotEP-1	0.902	0.912	0.898	0.905	0.899	0.921	1.000	0.972	0.992	0.455	0.452	0.300	0.539	0.590	0.603	0.384	0.414	0.413
cotEP-2	0.828	0.842	0.841	0.828	0.824	0.857	0.972	1.000	0.978	0.504	0.509	0.346	0.576	0.628	0.630	0.452	0.479	0.484
cotEP-3	0.914	0.922	0.915	0.919	0.913	0.933	0.992	0.978	1.000	0.444	0.445	0.293	0.531	0.578	0.592	0.377	0.403	0.404
emAX-SAM-2	0.340	0.359	0.374	0.316	0.314	0.351	0.455	0.504	0.444	1.000	0.975	0.853	0.833	0.783	0.818	0.722	0.659	0.700
emAX-SAM-3	0.330	0.349	0.372	0.302	0.303	0.339	0.452	0.509	0.445	0.975	1.000	0.854	0.853	0.810	0.828	0.769	0.739	0.765
emAX-SAM-4	0.202	0.217	0.225	0.183	0.179	0.203	0.300	0.346	0.293	0.853	0.854	1.000	0.656	0.589	0.621	0.559	0.502	0.546
emAX-PL-1	0.418	0.432	0.455	0.399	0.402	0.430	0.539	0.576	0.531	0.833	0.853	0.656	1.000	0.960	0.970	0.818	0.805	0.820
emAX-PL-2	0.450	0.471	0.489	0.427	0.437	0.468	0.590	0.628	0.578	0.783	0.810	0.589	0.960	1.000	0.979	0.821	0.846	0.836
emAX-PL-3	0.484	0.503	0.516	0.465	0.471	0.501	0.603	0.630	0.592	0.818	0.828	0.621	0.970	0.979	1.000	0.777	0.780	0.783
emAX-RT-1	0.233	0.252	0.283	0.202	0.214	0.241	0.384	0.452	0.377	0.722	0.769	0.559	0.818	0.821	0.777	1.000	0.952	0.988
emAX-RT-2	0.257	0.282	0.308	0.220	0.242	0.271	0.414	0.479	0.403	0.659	0.739	0.502	0.805	0.846	0.780	0.952	1.000	0.980
emAX-RT-3	0.248	0.270	0.300	0.215	0.230	0.259	0.413	0.484	0.404	0.700	0.765	0.546	0.820	0.836	0.783	0.988	0.980	1.000
emAX-EPD-2	0.591	0.612	0.605	0.588	0.595	0.628	0.712	0.736	0.705	0.707	0.683	0.506	0.721	0.729	0.724	0.745	0.689	0.726
emAX-EPD-3	0.488	0.513	0.519	0.469	0.483	0.518	0.642	0.682	0.629	0.730	0.751	0.536	0.805	0.830	0.796	0.841	0.844	0.849
[illegible]	[illegible]	[illegible]	[illegible]	[illegible]	[illegible]	[illegible]	[illegible]	[illegible]	[illegible]	[illegible]	[illegible]	[illegible]	[illegible]	[illegible]	[illegible]	[illegible]	[illegible]	[illegible]

Table S2. Comparison of median mRNA prevalences of TFs and subregion specific mRNAs to all detected mRNAs for each subregion. For each subregion, medians tested for equality are listed, with the Wilcoxon rank sum test p values. Statistically significant differences in the median (p<0.05) are in bold.

		Median			Median		
		**all mRNAs**	**TF mRNAs**	**p value**	**all mRNAs**	**Subregion Specific mRNAs**	**p value**
**glob**	**EP**	9.14	7.89	**1.24E-04**	9.14	13.93	**1.12E-04**
	**SUS**	11.09	8.71	**6.30E-08**	11.09	33.91	**5.04E-78**
	**ES**	8.60	7.08	**1.19E-05**	8.60	24.24	**2.06E-47**
	**SC-EPD**	9.23	9.10	5.67E-01	9.23	35.42	**2.77E-33**
	**SC-OI**	8.24	7.81	6.83E-01	8.24	16.36	**2.34E-15**
	**SC-II**	9.21	8.51	1.76E-01	9.21	24.54	**1.58E-25**
	**SC-ENT**	9.71	8.69	**2.00E-02**	9.71	30.21	**7.01E-18**
	**SC-HI**	8.63	8.24	4.34E-01	8.63	13.49	**9.70E-08**
**hrt**	**EP**	8.30	6.64	**1.23E-07**	8.30	9.81	**8.19E-06**
	**SUS**	9.62	7.93	**1.43E-05**	9.62	26.51	**1.35E-73**
	**ES**	6.77	4.70	**5.42E-16**	6.77	8.90	**1.03E-17**
	**SC-EPD**	9.05	8.37	1.29E-01	9.05	13.45	**3.91E-07**
	**SC-OI**	8.45	7.96	1.99E-01	8.45	11.67	**3.59E-10**
	**SC-II**	9.50	8.24	**6.42E-03**	9.50	33.47	**2.57E-51**
	**SC-ENT**	9.71	8.31	**3.56E-04**	9.71	19.95	**8.26E-13**
	**SC-HI**	8.68	7.45	**5.69E-04**	8.68	8.66	**2.31E-02**
**cot**	**EP**	8.13	6.27	**1.46E-09**	8.13	10.84	**9.37E-12**
	**SUS**	9.96	8.25	**2.08E-04**	9.96	27.39	**2.08E-75**
	**ES**	7.25	5.64	**1.95E-07**	7.25	12.02	**1.74E-27**
	**SC-EPD**	9.21	7.61	**7.88E-06**	9.21	16.51	**2.81E-07**
	**SC-OI**	8.88	6.94	**2.29E-07**	8.88	13.11	**1.79E-22**
	**SC-II**	9.10	7.63	**3.13E-04**	9.10	31.52	**4.65E-45**
	**SC-ENT**	9.25	7.32	**2.34E-06**	9.25	22.99	**1.51E-08**
	**SC-HI**	8.59	7.27	**8.70E-04**	8.59	13.99	**2.08E-06**
**em**	**AX-SAM**	10.96	10.75	6.84E-01	10.96	76.31	**5.82E-11**
	**AX-PL**	8.36	7.77	**4.54E-02**	8.36	19.69	**2.75E-02**
	**AX-RT**	8.50	8.06	**1.28E-02**	8.50	13.63	**5.25E-03**
	**AX-EPD**	10.01	8.88	**1.19E-02**	10.01	41.83	**5.80E-06**
	**AX-PY**	9.42	8.23	**1.62E-03**	9.42	71.88	**4.11E-02**
	**AX-VS**	8.71	8.08	1.19E-01	8.71	12.68	2.02E-01
	**AX-ST**	9.62	9.38	6.10E-01	9.62	40.49	**4.56E-05**
	**COT-ADEPD**	10.04	9.53	2.85E-01	10.04	28.04	**5.29E-04**
	**COT-ABEPD**	9.79	8.74	**2.86E-02**	9.79	71.56	**4.48E-10**
	**COT-ADPY**	10.06	9.65	2.31E-01	10.06	50.47	4.50E-01
	**COT-ABPY**	10.48	9.87	6.81E-02	10.48	69.07	**1.29E-02**
	**COT-VS**	9.23	8.71	3.32E-01	9.23	21.47	**3.43E-03**
	**ES**	8.95	9.08	9.39E-01	8.95	20.38	**9.31E-45**
	**SC-PS**	9.86	8.74	**1.16E-02**	9.86	38.97	**6.37E-24**
	**SC-HG**	9.85	8.03	**5.06E-05**	9.85	77.09	**7.79E-70**
	**SC-PY**	8.46	7.77	**6.97E-03**	8.46	23.26	**2.22E-24**
	**SC-HI**	8.43	7.26	**2.36E-03**	8.43	24.83	**5.70E-21**

Supporting Information References

1. R. Kumar *et al.*, A high-throughput method for Illumina RNA-Seq library preparation. *Frontiers in Plant Science* **3** (2012).
2. Z. Lu, B. T. Hofmeister, C. Vollmers, R. M. DuBois, R. J. Schmitz, Combining ATAC-seq with nuclei sorting for discovery of cis-regulatory regions in plant genomes. *Nucleic Acids Res.* **45**, e41 (2017).
3. M. F. Belmonte *et al.*, Comprehensive developmental profiles of gene activity in regions and subregions of the Arabidopsis seed. *Proc Natl Acad Sci USA* **110**, E435-E444 (2013).
4. B. Zhang, S. Horvath, A general framework for weighted gene co-expression network analysis. *Stat Appl Genet Mol Biol* **4**, Article17 (2005).
5. P. Langfelder, S. Horvath, WGCNA: an R package for weighted correlation network analysis. *BMC Bioinformatics* **9**, 559 (2008).
6. M. I. Love, W. Huber, S. Anders, Moderated estimation of fold change and dispersion for RNA-seq data with DESeq2. *Genome Biol* **15**, 550 (2014).
